# The secreted protein Cowpox Virus 14 contributes to viral virulence and immune evasion by engaging Fc-gamma-receptors

**DOI:** 10.1371/journal.ppat.1010783

**Published:** 2022-09-19

**Authors:** Ravi F. Iyer, David M. Edwards, Philipp Kolb, Hans-Peter Raué, Chris A. Nelson, Megan L. Epperson, Mark K. Slifka, Jeffrey C. Nolz, Hartmut Hengel, Daved H. Fremont, Klaus Früh

**Affiliations:** 1 Vaccine and Gene Therapy Institute, Oregon Health and Science University, Beaverton, Oregon, United States of America; 2 Institute of Virology, Medical Center, Faculty of Medicine, University of Freiburg, Freiburg, Germany; 3 Oregon National Primate Research Center, Oregon Health & Science University, Beaverton, Oregon, United States of America; 4 Department of Pathology & Immunology, Washington University School of Medicine in St. Louis, St. Louis, Missouri, United States of America; 5 Department of Molecular Microbiology and Immunology, Oregon Health & Science University, Portland, Oregon, United States of America; 6 Department of Biochemistry & Molecular Biophysics, Washington University School of Medicine in St. Louis, St. Louis, Missouri, United States of America; 7 Department of Molecular Microbiology, Washington University School of Medicine in St. Louis, St. Louis, Missouri, United States of America; University of Zurich, SWITZERLAND

## Abstract

The genome of cowpoxvirus (CPXV) could be considered prototypical for orthopoxviridae (OXPV) since it contains many open reading frames (ORFs) absent or lost in other OPXV, including vaccinia virus (VACV). These additional ORFs are non-essential for growth *in vitro* but are expected to contribute to the broad host range, virulence and immune evasion characteristics of CPXV. For instance, unlike VACV, CPXV encodes proteins that interfere with T cell stimulation, either directly or by preventing antigen presentation or co-stimulation. When studying the priming of naïve T cells, we discovered that CPXV, but not VACV, encodes a secreted factor that interferes with activation and proliferation of naïve CD8+ and CD4+ T cells, respectively, in response to anti-CD3 antibodies, but not to other stimuli. Deletion mapping revealed that the inhibitory protein is encoded by CPXV14, a small secreted glycoprotein belonging to the poxvirus immune evasion (PIE) family and containing a smallpoxvirus encoded chemokine receptor (SECRET) domain that mediates binding to chemokines. We demonstrate that CPXV14 inhibition of antibody-mediated T cell activation depends on the presence of Fc-gamma receptors (FcγRs) on bystander cells. *In vitro*, CPXV14 inhibits FcγR-activation by antigen/antibody complexes by binding to FcγRs with high affinity and immobilized CPXV14 can trigger signaling through FcγRs, particularly the inhibitory FcγRIIB. *In vivo*, CPXV14-deleted virus showed reduced viremia and virulence resulting in reduced weight loss and death compared to wildtype virus whereas both antibody and CD8+ T cell responses were increased in the absence of CPXV14. Furthermore, no impact of CPXV14-deletion on virulence was observed in mice lacking the inhibitory FcγRIIB. Taken together our results suggest that CPXV14 contributes to virulence and immune evasion by binding to host FcγRs.

## Author summary

Coxpoxvirus is considered the prototypical member of the closely related orthopox virus family that includes monkeypoxvirus and Variola major which caused smallpox. Presumably due to the presence of a large number of ORFs encoding for immunomodulators, CPXV is capable of infecting a wide variety of host species. Here, we show that the ORF CPXV14 encodes a small secreted protein that binds to host receptors for the constant Fc region of IgG antibodies thus preventing the ability of antibodies to engage these receptors. Viruses lacking CPXV14 are less virulent in animals but elicit increased immune responses suggesting that the interference with antibody function contributes to the ability of CPXV to evade immune control. Targeting Fc receptors is thus a new type of immune evasion mechanism that has possible applications for antibody mediated therapies.

## Introduction

The genus Orthopoxviridae (OPXV) is a group of large double-stranded DNA viruses that includes Variola (VARV), the causative agent of smallpox [1]. Although smallpox has been eradicated, there is a continued risk for accidental or deliberate reintroduction. In addition, several OPXV family members, including monkeypoxvirus (MPXV), can cause zoonotic infections resulting in occasional outbreaks [2]. Cowpoxvirus (CPXV) virus is one such zoonotic member of the OPXV [3]. Multiple clades of CPXV have been characterized [4] that occur naturally in rodents, occasionally making the jump into farm animals, pets and people [5]. Among the OPXV, CPXV possesses the largest genome including a number of non-essential, mostly uncharacterized "accessory”ORFs that are expected to contribute to the broad host tropism, virulence and immune evasion of CPXV [6]. It is assumed that a CPXV-like ancestor gave rise to VARV and other OPXV that display a very limited host tropism most likely due to the loss of tropism and immune evasion ORFs that is reflected in shorter genomes [1].

The broad host tropism of CPXV, including laboratory mice, together with the full complement of host-modulating ORFs renders CPXV an excellent model to study the role of viral immune modulation for infection and virulence of complex, large DNA viruses [7–9]. Many host modulatory ORFs are shared between CPXV and the most commonly studied, but highly passaged and attenuated, vaccinia virus (VACV), the horsepoxvirus-derived smallpox vaccine [10]. In addition, CPXV contains multiple putative immune evasion proteins that are shared to a varying degree with other OPXV but absent from VACV [11]. Among CPXV-specific ORFs are CPXV12 and CXPXV203 that prevent the stimulation of CD8+ T cells by inhibiting antigen presentation by major histocompatibility complex class I (MHC-I) either retaining MHC-I in the endoplasmic reticulum (ER) [12–15] or by blocking TAP-dependent peptide transport into the ER [16,17]. Due to MHC-I inhibition, CPXV elicits CD8+ T cell responses by cross-priming [8] compared to direct priming of VACV-specific CD8+ T cells [18] and MHC-I inhibitors allow CPXV to evade CD8+ T cell recognition *in vivo* [9]. CD4+ T cells are not affected by these inhibitors of antigen presentation. Instead, their stimulation is directly inhibited by CPXV219, a B22 family protein [19]. The related protein MPXV197 in MPXV additionally inhibits CD8+ T cells and MPXV lacking MPXV197 is highly attenuated *in vivo* [19]. In addition, CPXV encodes soluble proteins that interfere with co-stimulation of T cells or activation of NK cells [7,20].

Here, we describe a novel function for the small secreted protein CPXV14 and its role in virulence and immune evasion. CPXV14 is a member of a superfamily of OPXV proteins which share the conserved structural poxviral immune evasion (PIE) domain [21]. Furthermore, CPXV14 is one of five CPXV proteins containing the smallpox encoded chemokine receptor (SECRET) domain which enables members of this protein family to bind and sequester a wide range of mouse and human CC and CXC chemokines [22,23]. We demonstrate that CPXV14 prevents *in vitro* activation and proliferation of naïve T cells in response to anti-CD3 antibodies. Since deletion of CPXV14 from the CPXV genome restored T cell activation it seems that this function is unique among CPXV-encoded SECRET-domain proteins. Further investigation revealed that CPXV14 interference with antibody-mediated T cell activation required the presence of Fc-gamma receptors (FcγR) on bystander cells. Moreover, CPXV14 directly bound to FcγRs and interfered with the antibody-mediated activation of FcγRs *in vitro*. CPXV lacking CPXV14 displayed reduced virulence and viremia in wildtype mice, but not in mice lacking the inhibitory FcγRIIB, while at the same time eliciting increased T cell and antibody responses. CPXV14-mediated interference with host FcγRs thus seems to contribute to virulence by dampening both T cell and B cell responses during infection.

## Results

### CPXV14 prevents antibody-mediated T cell activation

**Fig 1:**
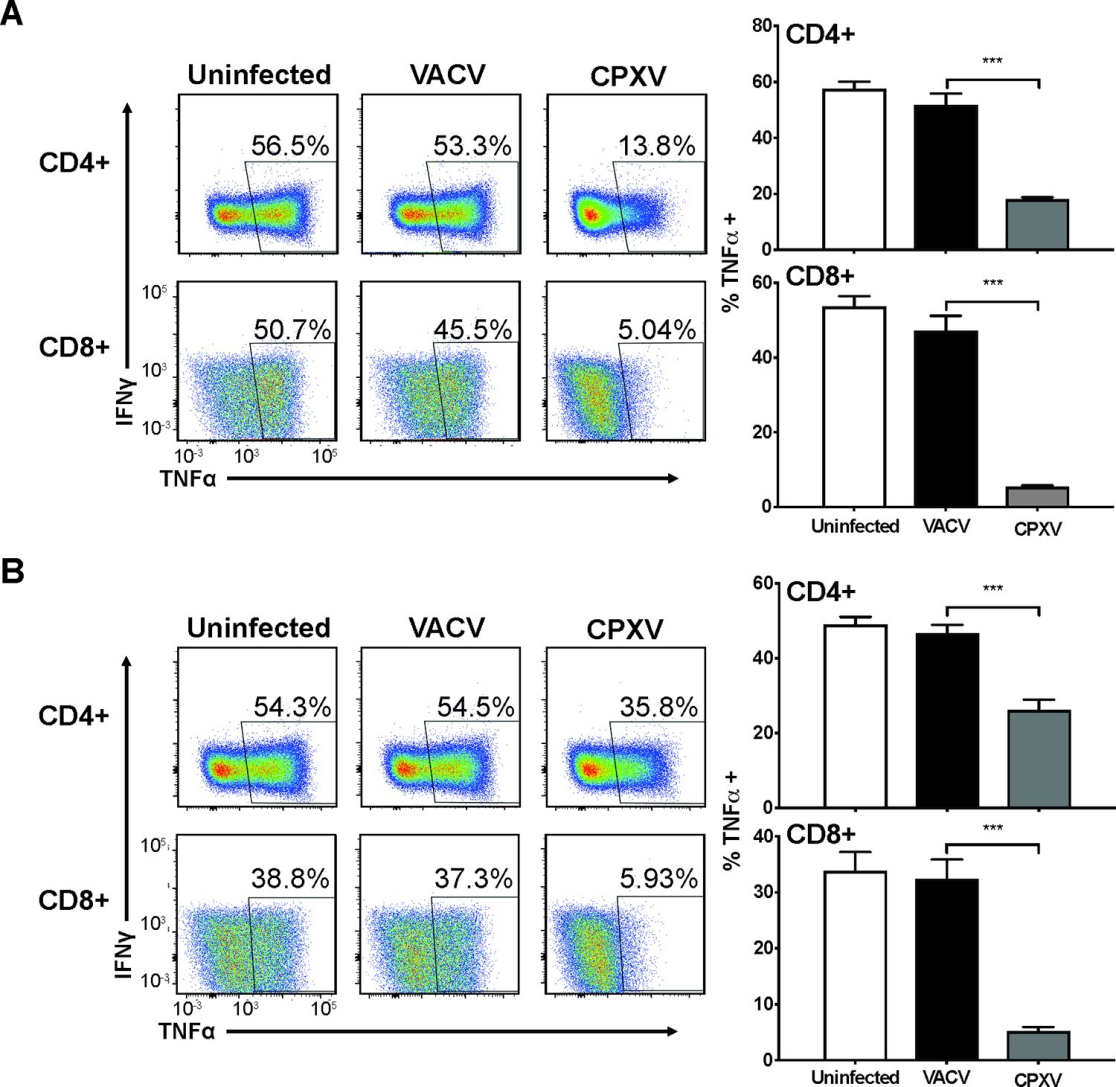
A secreted factor of CPXV prevents the activation of naïve T cells by anti-CD3 and anti-CD28. **A)** Reduced activation of naïve murine T cells by anti-CD3 and anti-CD28 in the presence of CPXV-infected cells. Murine A20 B cell lymphoma cells were infected at a multiplicity of infection (MOI) of 5 for 16h with CPXV or VACV, or uninfected prior to co-incubation with splenocytes from BALB/cByJ mice for 4h and transfer to plate-bound α-CD3ε and α-CD28 antibodies followed by ICS for IFNγ and TNFα (S1 Fig). **B)** CPXV-infected cells secrete a factor that prevents T cell activation. Murine MC57 fibrosarcoma cells were infected with CPXV, VACV (MOI = 5) or uninfected for 16h. Supernatants were harvested and incubated for 4h with splenocytes from BALB/cByJ mice followed by transfer to plate-bound α-CD3 and α-CD28 antibodies and ICS as in (A). Left: representative dot plots. Values represent the percentage of TNFα+ cells gated on live CD3+ CD4+ or CD8+ lymphocyte single cells. Right: Average frequencies of TNFα+ CD4+ or CD8+ T cells (+SEM) of 3 individual experiments each with 5 mice. Error bars indicate the Standard Error of Mean (SEM), p-values were calculated using unpaired two-tailed Student’s T test (***p≤0.001).

Since CPXV was shown to prevent stimulation of poxvirus-specific memory T cells, either by direct inhibition of TCR signaling or by interfering with antigen presentation, we were wondering whether CPXV additionally interferes with the antigen-independent activation of naïve T cells by antibodies. Therefore, we co-incubated murine A20 B cell lymphoma cells that were uninfected or infected overnight with CPXV (Brighton Red (BR) strain) or VACV (MOI = 5) (Western Reserve) with splenocytes from BALB/c mice together with plate bound α-CD3 and α-CD28 antibodies. T cell stimulation was monitored by intracellular cytokine staining (ICS) for TNFα and IFNγ (**S1 Fig**). As shown in **Fig 1A**, CPXV but not VACV inhibited the activation of naïve T cells by α-CD3 and α-CD28, with a more pronounced effect on CD8+ T cells.

To determine whether this inhibitory factor was secreted by CPXV-infected cells we prepared clarified supernatant (SN) from CPXV or VACV-infected MC57G fibroblasts. The SN was added at a 1:1 ratio to the culture media of splenocytes, after 2h the cells were transferred to α-CD3 and α-CD28 antibody coated plates and T cell simulation was monitored by ICS. As shown in **Fig 1B**, activation of both CD4+ and CD8+ T cells was inhibited by SN from CPXV-infected, but not VACV-infected cells, with CD8+ T cell inhibition again more pronounced compared to CD4+ T cells. These observations thus suggested that CPXV encodes a secreted factor that interferes with the activation of naïve T cells by plate-bound antibodies.

**Fig 2:**
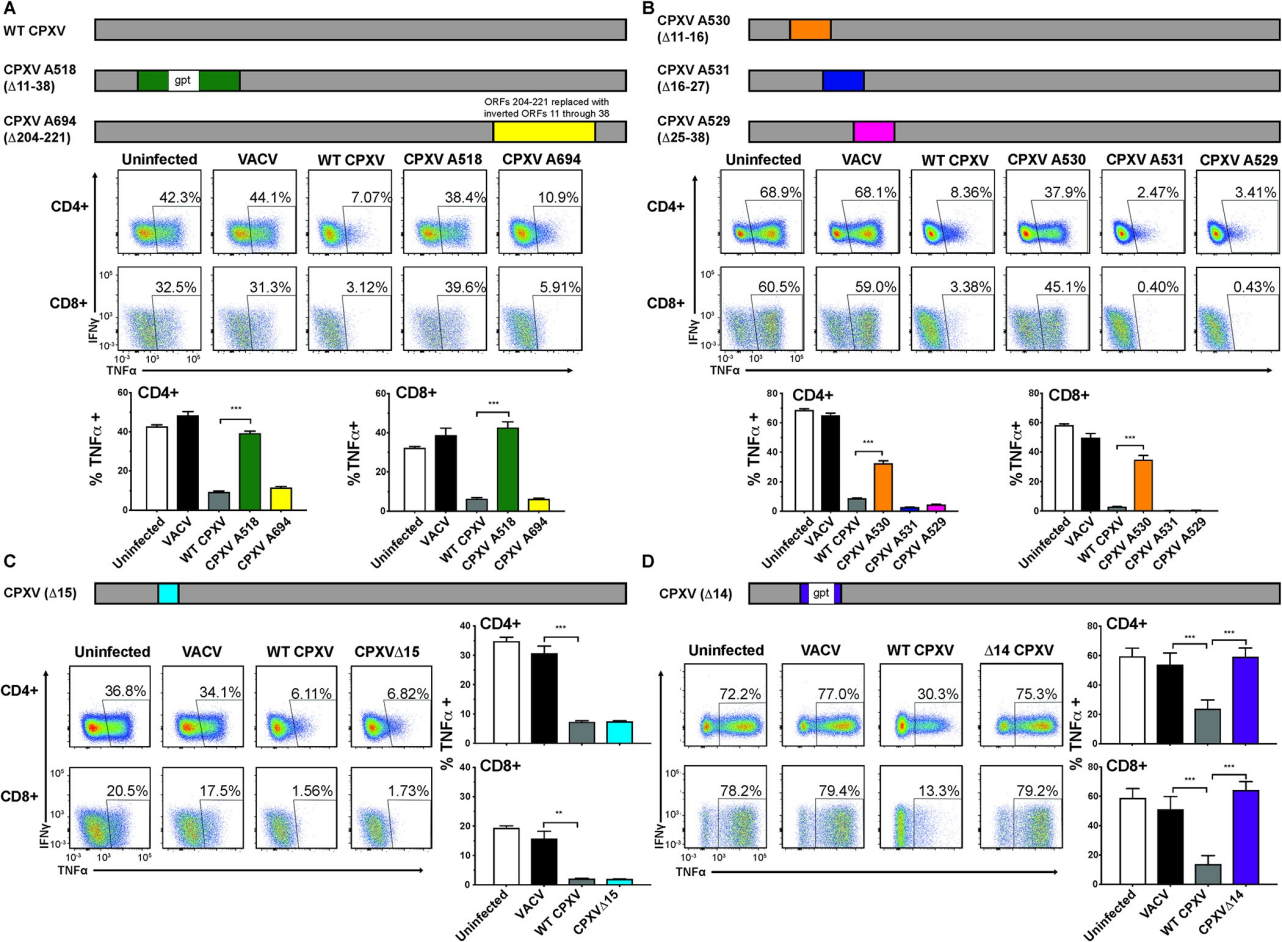
CPXV14 inhibits activation of naïve T cells by anti-CD3 and anti-CD28. **A)** A20 cells were infected with VACV, CPXV, or the two large deletion mutants A518 or A694 (MOI = 5), or uninfected and co-incubated with splenocytes from BALB/cByJ mice prior to α-CD3/α-CD28 stimulation and ICS as described in Fig 1. Top: Schematic of the approximate size and location of the deletion, Middle: dot plot from a representative experiment. Bottom: Average frequencies of TNFα+ CD4+ or CD8+ T cells (+SEM) within each experimental group (n = 5). **B)** A20 cells were infected with the deletion viruses A530, A531 or A529 (MOI = 5) or control viruses prior to co-incubation with splenocytes from BALB/cByJ mice, followed by α-CD3/α-CD28 stimulation and ICS. Bottom: Average frequencies of TNFα+ CD4+ or CD8+ T cells (+SEM) within each experimental group (n = 5). **C)** A20 cells were infected with CPXVΔ15, VACV or CPXV (MOI = 5), or uninfected prior to co-incubation with splenocytes from BALB/cByJ mice, α-CD3/α-CD28 stimulation and ICS. Right panel: Average frequencies of TNFα+ CD4+ or CD8+ T cells (+SEM) within each experimental group (n = 5). **D)** CPXVΔ14 was generated and characterized as shown in S2 Fig. A20 cells infected with CPXVΔ14 or control viruses (MOI = 5) were co-incubated with splenocytes from BALB/cByJ mice followed by α-CD3/α-CD28 stimulation and ICS. Right Panel: Average frequencies of TNFα+ CD4+ or CD8+ T cells (+SEM) from two experiments each with n = 5 per experimental group. Indicated P-values were calculated using unpaired two-tailed Student’s T test.

To identify the specific open reading frame (ORF) responsible for inhibiting T cell activation we initially screened two previously described CPXV recombinants with large deletions in the non-essential regions encoded in the terminal regions of the genome. CPXV A694 lacks 33.7 kb at the right end of the genome with the deleted region replaced by an inverted copy of the left-hand end of the genome [19] whereas CPXV A518 lacks 28.5 kb in the left end of the genome [16] (**Fig 2A**). When A20 cells infected with VACV, CPXV, A694 or A518 were added to splenocytes activated by plate-bound α-CD3 and α-CD28 antibodies we observed that CPXV and A694, but not VACV and A518 reduced CD4+ and CD8+ T cell activation (**Fig 2A**). Since these results suggested that the inhibitory factor was encoded in the left end of the genome we examined T cell inhibition by three overlapping deletion mutants lacking ORFs 11–16 (A530), ORFs 16–27 (A531) or ORFs 25–38 (A529) (**Fig 2B**). Whereas CPXVΔ16–27 (A531) and CPXVΔ25–38 (A529) interfered with antibody-mediated CD4+ and CD8^+^ T cell activation in splenocytes cultures, deletion of CPXV11-16 restored T cell activation upon co-incubation with infected A20 cells (**Fig 2B**). This result narrowed down the possible candidates to the five ORFs CPXV11 to CPXV15. Only two of these five ORFs encoded proteins predicted to be secreted: CPXV15 and CPXV14. CPXV15 was a possible candidate for the T cell inhibitory factor since this ORF encodes a soluble TNF receptor (TNFR) family protein with homology to CD30 [24]. Moreover, the ectromelia virus (ECTV) homolog of CPXV15 was previously shown to block IFNγ production by T cells [25]. Using the previously generated ORF15-deletion mutant A624 [24] we examined the ability of CPXV15 to prevent Ab-mediated T cell activation. However, when co-incubated with α-CD3 and α-CD28 activated splenocytes, A624 still inhibited CD4+ and CD8+ T cell activation thus ruling out ORF15 as the responsible ORF (**Fig 2C**).

The remaining candidate, CPXV14, belongs to a superfamily of proteins that share sequence and structural features termed the poxvirus immune evasion (PIE) domain [21]. Moreover, CPXV14 also contains a smallpox virus-encoded chemokine receptor (SECRET) domain that was shown to bind to a subset of CC and CXC chemokines [22]. To determine whether CPXV14 was responsible for the observed T cell inhibition we deleted the CPXV14 ORF from CPXV by homologous recombination replacing CPXV14 with a GFP-GPT cassette (**S2A Fig**). The recombinant virus was plaque purified three times under gpt selection conditions (**S2B Fig).** Using next-generation sequencing of the entire viral genome we demonstrated that no other CPXV ORF was affected by the *in vivo* homologous recombination (**S2C Fig**). CPXV14 is expressed with late kinetics and deletion of CPXV14 did not affect viral growth in vitro (**S3 Fig**). When CPXVΔ14-infected cells were added to α-CD3/α-CD28-stimulated splenocytes, we no longer observed a reduction of stimulation as observed for wildtype CPXV. Instead, CD4+ and CD8+ T cell stimulation was unaffected by CPXVΔ14-infected cells similar to VACV (**Fig 2D**). These observations suggested that CPXV14 was necessary for the observed inhibition of T cell activation.

**Fig 3:**
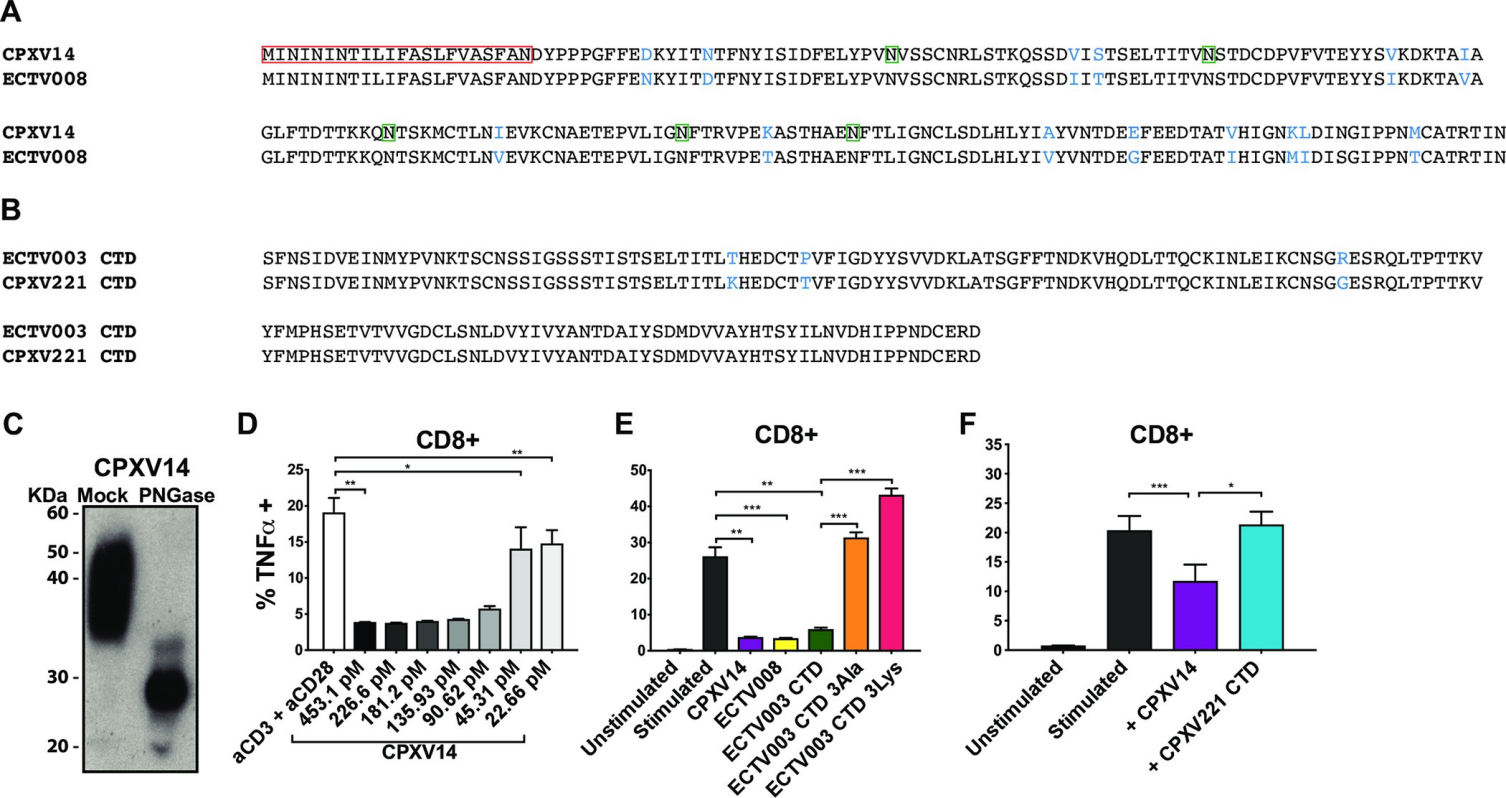
Poxviral SECRET-domain proteins inhibit T cell activation by plate-bound anti-CD3 and anti-CD28 antibodies. **A)** Alignment of CPXV14 with SECRET-domain containing protein-2 (ECTV008) of ectromelia virus (ECTV). Proteins were aligned using CLUSTAL O v1.2.1. The predicted signal sequence is highlighted with a red box. N-linked glycosylation sites were predicted with GlycolEP and are indicated in green. Amino-acid differences are shown in blue. **B)** Alignment of the SECRET-domain containing C terminal domain (CTD) of CPXV221 (CrmD) with that of the CrmD CTD in ECTV003. **C)** N-linked glycosylation of CPXV14-His. Immunoblot of purified, recombinant CPXV14-His without or with PNGase treatment. **D)** Recombinant CPXV14-His inhibits T cell activation by plate bound antibodies. Splenocytes from BALB/cByJ mice were incubated with recombinant CPXV14-His at the indicated concentrations for 4h prior to ICS. The mean TNFα response frequencies of CD8+ T cells from 5 mice are shown (+SEM). **E)** T cell inhibition by ECTV homologs of CPXV14 and CPXV221 is dependent on an intact SECRET domain. The indicated recombinant proteins were added to splenocytes of BALB/cByJ mice at a concentration of 1.1μM for 4h prior to ICS as in (C). Average frequencies of TNFα+ CD8+ T cells are shown (n = 5, +SEM). **F)** The CTD of CPXV221 (CrmD) does not inhibit T cell activation. T cell inhibition by 1.1μM of CPXV14-His or his-tagged recombinant CTD of CPXV221 was examined as in (D). Average frequencies of TNFα+ CD8+ T cells are shown (3 experiments, n = 3 each, +SEM). P values in D, E and F were calculated using paired two-tailed Student’s T test (***p≤0.001,**p≤0.01, *p≤0.05, NS = p>0.05).

CPXV14 comprises 202 amino acids (AA) including a predicted 23 AA signal sequence and, upon cleavage, a predicted molecular weight (MW) of 22.5 kDa (**Fig 3A**). In addition, CPXV14 contains five predicted N-linked glycosylation sites. (**Fig 3A**). To determine whether purified CPXV14 would block T cell activation, we isolated a His-tagged version of CPXV14 from transfected HEK293F cells. The purified protein is highly glycosylated as shown by PNGase F treatment (**Fig 3C**). When increasing concentrations of purified CPXV14 were added to splenocytes in α-CD3/α-CD28 coated plates we observed that CD8+ T cell stimulation was reduced with a half-maximal concentration of 90pM (**Fig 3D**. Thus, CPXV14 is both necessary and sufficient for the observed inhibition of T cell stimulation by CPXV.

Inhibition of CD8+ T cell activation was also observed when SECRET-domain containing protein-2 (ECTV008), the ECTV homologue of CPXV14 (**Fig 3A**), was used in this assay (**Fig 3E**). SECRET domains are also found in the C-terminal domain (CTD) of the cytokine-response modifier (Crm) B and D proteins of CPXV and ECTV whereas the N-terminal domain contains TNF-binding activity [22,23]. To determine whether the observed inhibition of T cell stimulation was a general activity of SECRET domains, we isolated His-tagged versions of the ECTV003 CrmD CTD, including mutants that lacked a functional SECRET domain (3Ala-His, 3Lys-His) as well as the CrmD CTD of CPXV221 (**Fig 3B**). Interestingly, inhibition of CD8+ T cell stimulation was observed for the ECTV003 CrmD CTD (**Fig 3E**), but not for the CrmD CTD of CPXV221 (**Fig 3F)**, despite their close homology (**Fig 3B**). The two mutant proteins of ECTV003 CrmD CTD were also unable to inhibit T cell stimulation (**Fig 3E**). With the caveat that we did not examine the full-length protein of ECTV003, these observations suggested that ECTV encodes at least two SECRET-domain proteins capable of interfering with antibody-mediated T cell activation. However, it seems that CPXV14 is the only SECRET-domain containing protein encoded by CPXV capable of this interference given the finding that the knockout virus restores T cell stimulation.

### CPXV14 binds to Fcγ-receptors and interferes with antibody-mediated activation of T cells

**Fig 4:**
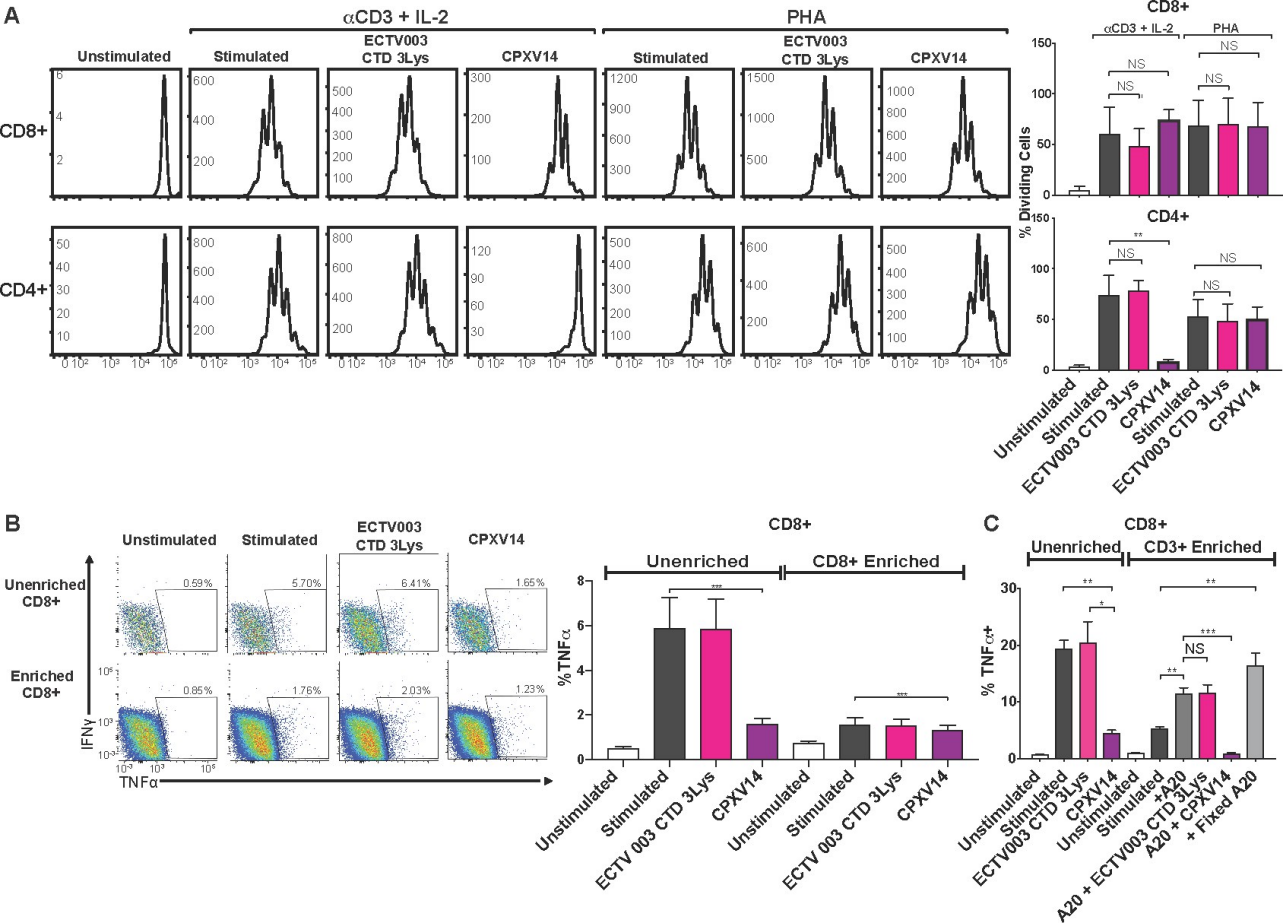
T cell inhibition by CPXV14 is limited to antibody stimulation and requires the presence of auxiliary cells. **A)** CPXV14 inhibits T cell proliferation when stimulated by α-CD3 + IL-2 but not by PHA. Splenocytes from BALB/cByJ mice were loaded with CFSE and stimulated with α-CD3 and recombinant murine IL-2 or PHA for 3 days in the presence or absence of the indicated recombinant proteins (100nM) followed by flow cytometry. Representative histograms collected from one animal (left) or average percentage (+SEM) of dividing cells from 5 mice (right) are shown. **B)** Reduced stimulation and lack of CPXV14 inhibition upon enrichment of CD8+ T cells. CD8+ T cells were enriched (>97% CD8+) or not from murine splenocytes and incubated with 100nM of CPXV14 or control protein (ECTV003 CTD-3Lys), and subsequently stimulated with plate-bound α-CD3 and α-CD28 followed by ICS. Left: Representative plots from one mouse. Right: Average frequencies of TNFα producing CD8+ T cells from 10 mice (2 experiments, n = 5 each) (+SEM). **C)** Stimulation and CPXV14 inhibition of enriched CD8+ T cells is restored in the presence of auxiliary cells. Splenocytes were either left unenriched or enriched to >85% CD3+ cells. Where indicated, live or fixed A20 cells, a BALB/c mouse-derived B cell line, were added to CD8+ T cells at a ratio of 1:2 (A20:CD3+) in the presence or absence of the indicated poxviral proteins (100 nM). Average frequencies of TNFα producing CD8+ T cells are shown (n = 5, +SEM). P-values were calculated using paired two-tailed Student’s T test (***p≤0.001,**p≤0.01, *p≤0.05, NS = p>0.05).

To investigate whether CPXV14 also impairs T cell proliferation we monitored T cell division after stimulation with soluble α-CD3 and IL-2 in the presence or absence of CPXV14, or a control protein (ECTV003 CrmD CTD 3Lys). Upon CFSE labeling, proliferation was monitored using flow cytometry. Untreated, or control-protein treated T cells proliferated in response to α-CD3 and IL-2 treatment as reflected in reduced CFSE fluorescence (**Fig 4A**). In the presence of CPXV14, however, proliferation of CD4+ T cells was highly reduced whereas CD8+ T cell proliferation was not affected in this assay. To determine whether antibody-independent T cell stimulation would be inhibited, we used phytohaemagglutinin (PHA) instead of α-CD3 to monitor proliferation in response to IL-2. However, CPXV14 did not impact PHA-mediated proliferation of CD4+ T cells (**Fig 4A**). Similarly, we did not observe inhibition when T cells were simulated by PMA/Ionomycin (**S4A Fig).** Since this observation raised the question whether CPXV14 interference only occurred when T cells were activated by α-CD3, but not by antigen-specific activation, we further examined whether the SIINFEKL peptide /Kb-dependent activation of naïve T cells obtained from OT-1 TCR transgenic mice would be inhibited by CPXV14. As with PHA and PMA, we did not observe inhibition (**S4B and S4C Fig**).

**Fig 5:**
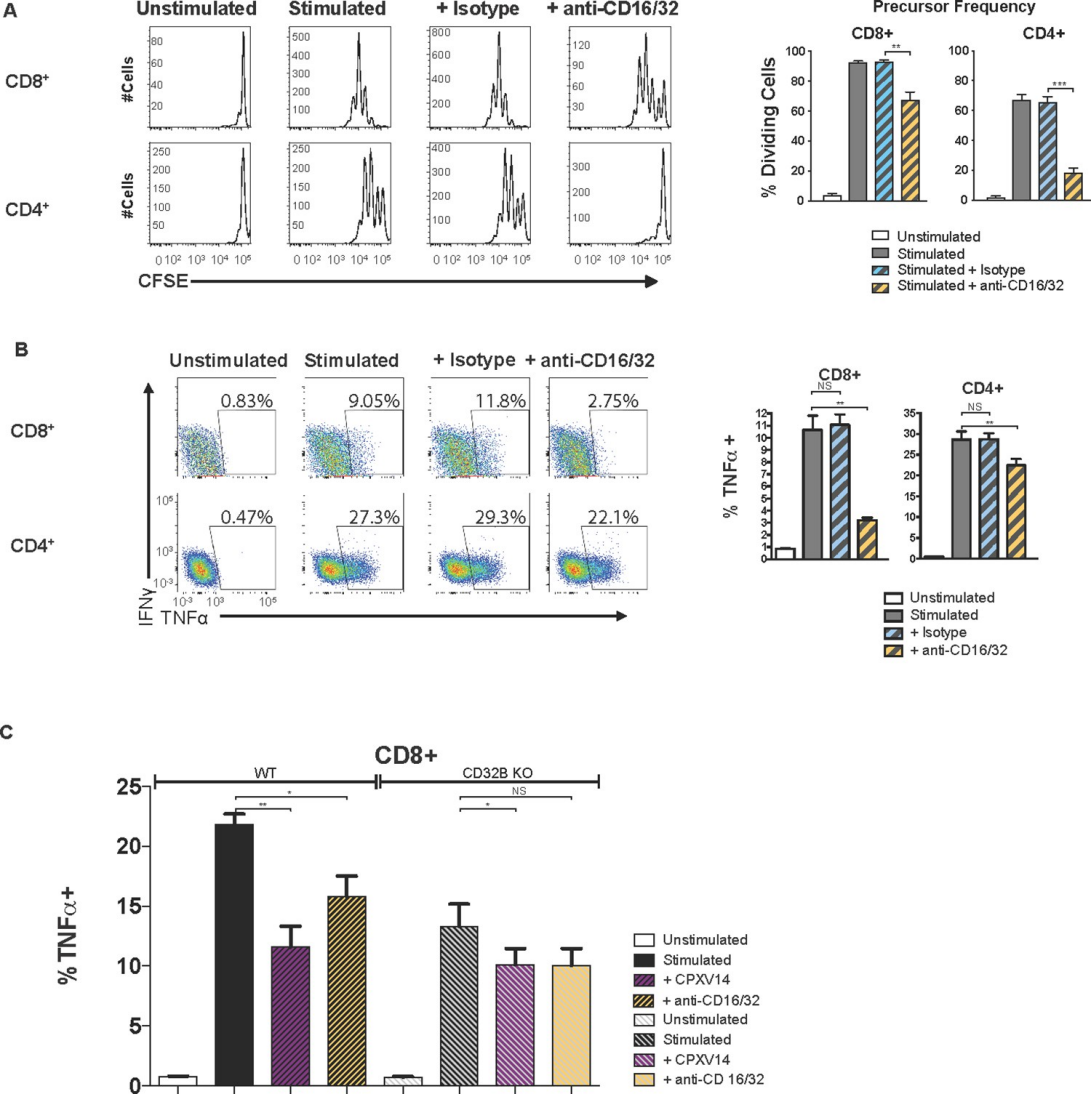
Inhibition of antibody-mediated T cell activation by CPXV14 depends on Fcγ-receptors on auxiliary cells and can be recapitulated with FcγR-targeting antibodies. **A)** FcγR antibodies inhibit proliferation of naïve T cells similar to CPXV14. Splenocytes from BALB/cByJ mice, unenriched or enriched for CD3+ cells (>98%), were loaded with CFSE and stimulated with α-CD3 and recombinant murine IL-2 for 3 days in the presence or absence of 200nM α-mCD16 and α-mCD32 followed by flow cytometry. Representative histograms for CD8+ and CD4+ T cells collected from one animal (left) or average percentage (+SEM) of dividing cells from 5 mice (right) are shown. **B)** FcγR antibodies inhibit activation of naïve T cells similar to CPXV14. Splenocytes from BALB/cByJ mice, unenriched or enriched for CD3+ cells, were incubated with 20nM α-mCD16/32 where indicated for 4h prior to ICS. Representative dot plots are shown on the left and the average TNFα+ response frequencies of T cells from 5 mice (+SEM) is shown on the right. **C)** Inhibition of antibody-mediated naïve T cell activation by CPXV14 is dependent on the low affinity FcγRIIB (CD32B). Splenocytes from three CD32B KO mice and three B6129SF2/J control mice were incubated with 100nM of CPXV14-His or the α-mCD16/32 where indicated for 4h prior to ICS. The average frequencies of TNFα+ CD8+ T cells (+SEM) are shown. P-values were calculated using unpaired two-tailed Student’s T test (***p≤0.001,**p≤0.01, *p≤0.05, NS = p>0.05).

Together, these observations suggested that CPXV14 interfered only with antibody-mediated activation of naïve T cells. To determine whether this was due to CPXV14 interfering with α-CD3 binding to T cells, we purified CD8+ T cells prior to Ab-dependent stimulation. Surprisingly, purified CD8+ T cells responded poorly to antibody-mediated T cell stimulation, even when plate-bound. Moreover, CPXV14 was less able to prevent this residual stimulation (**Fig 4B**). In contrast, CD8+ T cell stimulation and CPXV14 inhibition was restored in the presence of A20 B cell lymphoma bystander cells (**Fig 4C**). The requirement for accessory cells for α-CD3-mediated T cell proliferation has been reported previously [26–28]. Moreover, it was reported that this auxiliary effect was due to Fcγ-receptors (FcγR) expressed by bystander cells [26,29,30]. Indeed, when a mixture of FcγR-targeting antibodies (anti-FcγRIII (CD16) and anti-FcγRIIB (CD32)) was added to plate-bound α-CD3 and α-CD28, we observed a significant reduction of T cell proliferation (**Fig 5A**) and T cell stimulation (**Fig 5B**). Similar to CPXV14, inhibition of CD4+ proliferation was more pronounced than inhibition of CD8+ T cell proliferation (**Fig 5A**) whereas the reverse was true for T cell stimulation by α-CD3/α-CD28 (**Fig 5B**). The similarity of the inhibitory effect of FcγR-targeting antibodies and CPXV14 on T cell stimulation by α-CD3 suggested that CPXV14 might inhibit T cell activation similarly by interference with the FcγR. Indeed, CD8+ T cell activation in splenocytes from FcγRIIB-knockout mice was less inhibited by CPXV14 compared to wildtype mice (**Fig 5C**). Since naïve T cells do not express FcγRIIB [31], we conclude that CPXV14 inhibited antibody-mediated T cell activation by preventing the FcγRIIB-dependent support by bystander cells.

**Fig 6:**
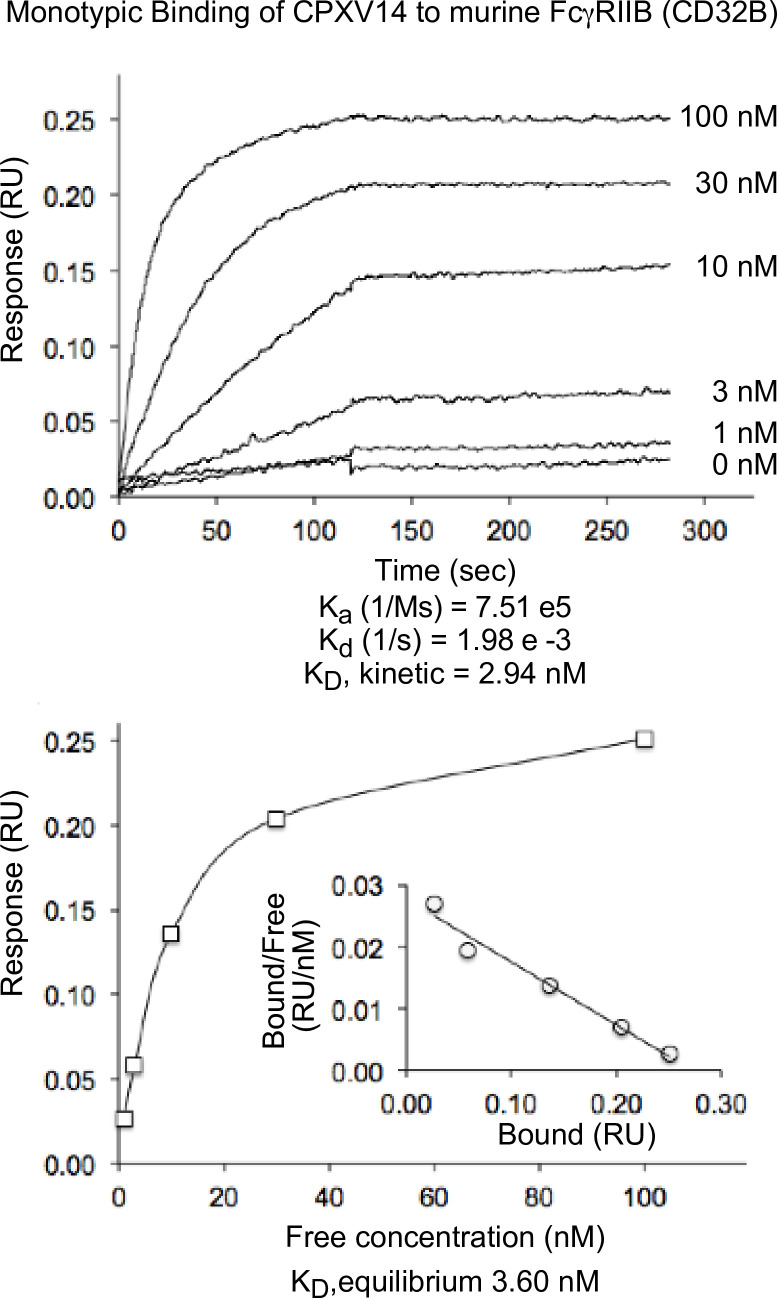
CPXV14 binds to murine FcγRIIB (mCD32B). Randomly biotin-labeled recombinant CPXV14-His was loaded onto streptavidin biosensors for Bio-Layer Interferometry (BLI) experiments. The pins were submerged in the indicated concentrations of soluble recombinant mCD32B ectodomain. Shown in the upper panel is the shift in wavelength measured over time due to optical interference. CPXV14 binds to murine FcγRIIB (mCD32B) with high affinity. A summary table of the affinity constants determined by kinetic analysis is given under the sensorgram, k_a_(1/Ms), k_d_(1/s), and K_D kinetic_(M). The K_D_ of CPXV14-His binding to this receptor was calculated to be 3.60 nM at steady state. The equilibrium concentration curves were fitted at steady-state assuming a 1:1 binding model.

To obtain direct measurements of CPXV14 binding to FcγRIIB we used Bio-Layer Interferometry (BLI). Recombinant CPXV14 was randomly biotinylated and loaded onto streptavidin pins. These pins were then submerged into a solution containing murine FcγRIIB (CD32B) (**Fig 6**). As shown, CPXV14 bound to murine FcγRIIB with 3.60nM nanomolar affinity. By comparison, the K_D_ of mFcγRIIB’s typical antibody ligands, mIgG1, mIgG2A, and mIgG2B, is roughly 100nM [32]. Thus, CPXV14 binds to FcγRIIB with high affinity.

**Fig 7:**
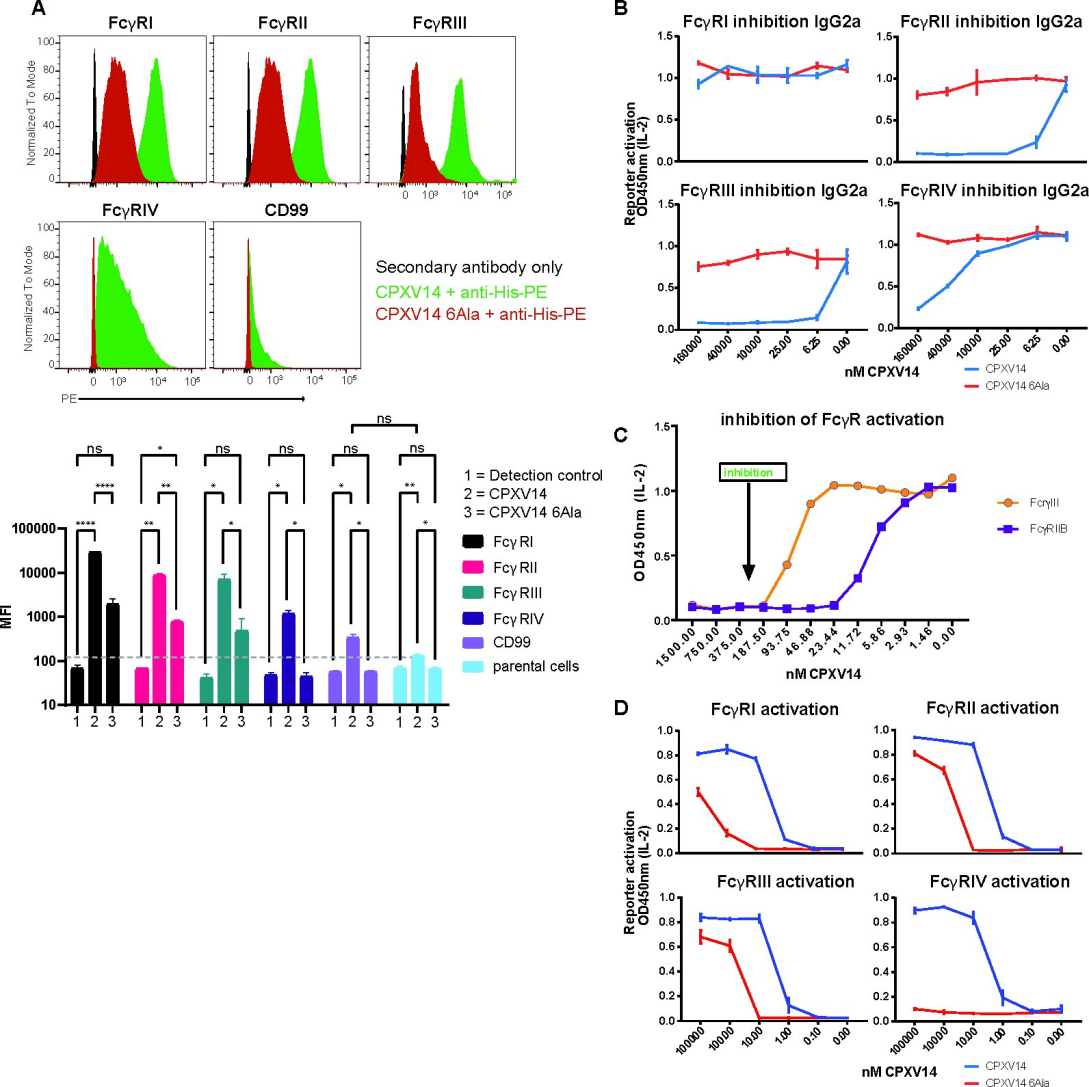
CPXV14 can act as an agonist of FcγRs and antagonist of IgG-dependent FcγR signaling. **A)** CPXV14 directly binds to mouse FcγRs on BW5147 reporter cells. His6-tagged CPXV14 or its 6Ala mutant were incubated with the indicated reporter cells and protein binding was detected via flow cytometry using a PE-conjugated TetraHis-specific antibody. Human CD99 expressing or parental BW1547 cells served as controls. The dashed line indicates background binding to parental cells. The bar graph shows the mean fluorescence intensities (MFI) from three independent experiments. Error bars = SD. 2-way ANOVA (Tukey) (***p≤0.001,**p≤0.01, *p≤0.05, ns = p>0.05). **B)** CPXV14 interferes with IgG-dependent FcγR signaling. IL-2 reporter cells expressing the indicated murine FcγRs were added to mouse IgG2A-coated plates in the presence or absence of the indicated concentrations of either CPXV14-His or CPXV14-6Ala-His. The mean and range from two independent experiments (n = 2 each) is shown. **C)** CPXV14 prevents FcγRIIB and FcγRIV signaling in response to immune complexes. IL-2 reporter cells expressing murine FcγRIV (mCD16) or FcγRIIB (mCD32B) were added to a plate coated with immune complexes (TNFα + anti-TNFα) in the presence of CPXV14-His. Complete inhibition of IL-2 secretion was observed at 187.5 nM and 23.44 nM, respectively. The mean of duplicate measurements is shown. **D)** Immobilized CPXV14 activates FcγRs. IL-2 reporter cells expressing the indicated murine FcγRs were added to plates coated with the indicated concentrations of CPXV14-His or CPXV14-6Ala-His. IL-2 secretion indicates direct activation of FcγR signaling in response to CPXV14 binding. The mean and range from two independent experiments (n = 2 each) is shown.

To further determine whether CPXV14 interfered with FcγR activation by antibodies we used a panel of reporter cell lines based on mouse BW5147 thymoma cells that express the ectodomains of murine FcγRI, FcγRIIB, FcγRIIII and FcγRIV fused to the CD3-ζ-chain signaling module. FcγR activation is thus measured by induction of mIL-2 secretion using a sandwich ELISA [33,34]. We initially examined whether CPXV14 or a mutant version of CPXV14 in which charged residues homologous to those shown for the ECTV-CrmD-CTD to be involved in ligand binding were replaced with alanine (CPXV14-6Ala) would bind to FcγRs expressed by these cell lines. When CPXV14 was incubated with BW5147 cells expressing each FcγR or, for control, CD99 we observed strong binding to FcγRI, II and III but less binding to FcγR-IV and only background binding to CD99 (**Fig 7A**). In contrast, binding of CPXV-6Ala was much reduced compared to CPXV14. When plates were coated with murine IgG2A, each of the FcγRs was activated as reflected by IL2 secretion (**Fig 7B**). Addition of CPXV14 completely prevented the antibody-mediated activation of FcγRIIB and FcγRIII above 6.25nM whereas CPXV14-6Ala was not inhibitory. The activation of FcγRIV was almost completely inhibited at the highest concentrations used (1600 nM) consistent with the weaker binding of CPXV14 to FcγRIV-expressing cells observed for this receptor. In contrast, FcγRI, which binds with higher affinity to Ab than the other receptors tested [35], was not inhibited at the CPXV14 concentrations used despite the fact that CPXV14 clearly bound to this receptor. We further monitored the interference of CPXV14 with FcγRIIB and FcγRIII-activation by immune complexes by coating plates with recombinant human TNFα and α-TNF (mouse IgG1) antibody complexes. Complete inhibition of FcγRIIB and FcγRIII-activation was observed at concentrations above 23nM and 185nM, respectively (**Fig 7C**). These data suggest that CPXV14 competes with antibodies and antigen/antibody complexes for binding to FcγRs, particularly FcγRIII and FcγRIIB, thus preventing their activation and ensuing signal transduction.

Downstream signaling of FcγRs occurs in response to receptor cross-linking by antibody/antigen complexes or by immobilized antibodies [35]. Since CPXV14 seems to compete for antibody binding to the FcγR we were wondering if immobilized CPXV14 would similarly be able to activate FcγR signaling. Therefore, we coated plates with either CPXV14 or the inactive 6Ala mutant at decreasing concentrations prior to adding FcγR-expressing reported cells. Interestingly, we observed FcγR-dependent IL2 release with CPXV14 in the absence of added antibodies (**Fig 7D**). Consistent with the residual binding observed in **Fig 7A**, the mutant CPXV14 protein also stimulated FcγRI, II and III, albeit at higher concentrations compared to CPXV14, but not FcγRIV. These data confirm that CPXV14 interacts directly with surface expressed FcγRs. Moreover, these data suggest that CPXV14 not only competes with IgG for binding to their receptors, but also potentially engages FcγRs and triggers signaling upon cross-linking.

### CPXV14 contributes to CPXV virulence in a FcγR-dependent manner

**Fig 8:**
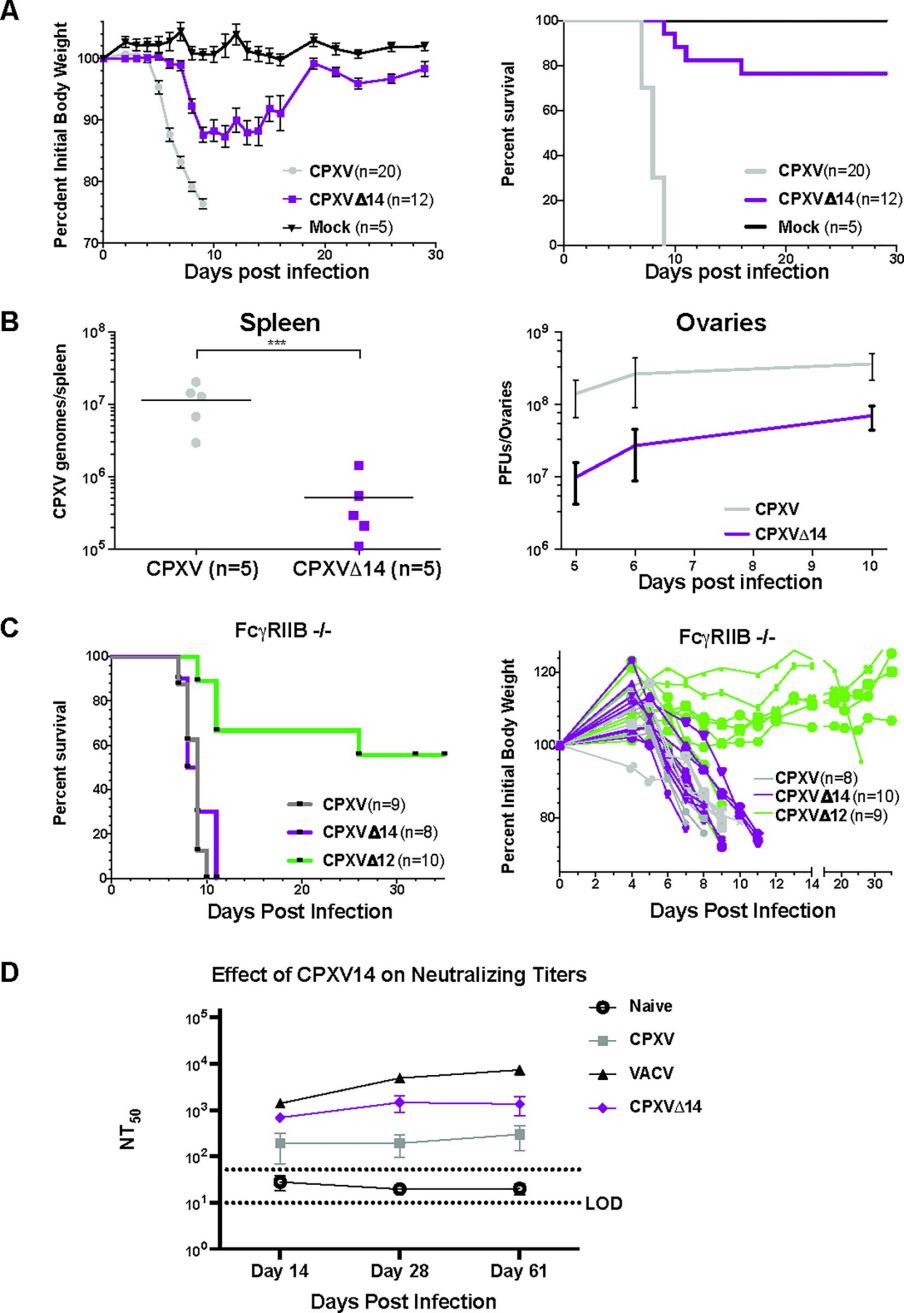
CPXV14 is a FcγRIIB-dependent virulence factor. **A)** Mice recover from lethal CPXV-infection in the absence of CPXV14. 11 week old female C57BL/6J mice were intranasally challenged with 2 x 10^5^ PFU of the indicated viruses or mock-injected and monitored for weight loss (left). Animals were sacrificed when weight loss exceeded 20%. Differences in survival curves (right) between CPXV and CPXVΔ14 were statistically significant (P< 0.0001) using Log-Rank (Mantel-Cox) test. The difference in mean weights of CPXV- and CPXVΔ14-infected mice were shown to be statistically significant on day 6 post-infection as determined by unpaired Student’s T test (P<0.0001). Shown are the combined results from 3 independent experiments. **B)** Reduced viral replication in the absence of CPXV14. **Left:** 11 week old female C57BL/6J mice were intranasally challenged with 2 x 10^5^ PFU of CPXV or CPXVΔ14. The total number of CPXV genome copies measured by qPCR in spleens of individual mice is shown at day 10 following infection. P-values of log-transformed data were calculated using two-tailed Student’s T test (P = 0.0003). **Right:** 11 week old female C57BL/6J mice were intraperitoneally challenged with 2 x 10^6^ PFU of CPXV or CPXVΔ14. Ovarian titers were calculated on days 5, 6 and 10 using standard plaque assay. For each time point, the combined data from the following number of animals, representing at least 3 independent experiments, are shown: day 5: CPXV (n = 9), CPXVΔ14 (n = 7); day 6: CPXV (n = 8), CPXVΔ14 (n = 7); day 10: CPXV (n = 9), CPXVΔ14 (n = 12). The titers were determined to be statistically different as determined using a 2-way ANOVA test (P = 0.0054). **C)** CPXVΔ14 regains virulence in FcγRIIB KO mice. FcγRIIB^-/-^(mCD32B) knockout mice (8–10 weeks old, female) on a C57BL/6 background were intranasally challenged with 9 x 10^5^ PFU of the indicated viruses or mock-injected and monitored for weight loss (left) and survival (right). Survival curves were found to be significantly different between CPXVΔ12 and CPXV or CPXVΔ14 (P = 0.002 and 0.0008, respectively) using Log-rank (Mantel-Cox) test. However, there was no statistical difference between the survival of mice infected with CPXV and CPXVΔ014 (P = 0.4154). Likewise, there was no statistically significant difference between the mean body weights of CPXV- and CPXVΔ14-infected mice on day 6 post-infection, as determined by unpaired Student’s T test (P = 0.6253). **D**) Deletion of CPXV14 results in reduced neutralizing Ab response. 11 week old female C57BL/6J mice were infected i.p. with 1x10^6^ PFU of indicated viruses (Naïve mice: n = 2 on days 14 and 28, n = 4 on day 61; all other groups: n = 5 on each day of harvest). Serum was collected at the indicated days. Neutralizing antibody titers were determined by incubating serial dilutions of serum with 80–100 PFU of CPXV for 2h at 37°C and determining remaining infectious virus by plaque assay. The 50% neutralizing titer (NT50) is shown as individual values with geometric mean (+/- 3x SD). The difference between CPXV and CPXVΔ14 was significant (p<0.0001) as shown by two-way ANOVA, with Tukey’s post test, of the mean neutralizing titers over time (using the log transformed data).

To elucidate the role of CPXV14 in virulence we infected mice intra-nasally with CPXV or CPXVΔ14 and monitored weight loss and survival. For control, mice were injected with phosphate buffered saline (PBS). All CPXV-infected mice infected displayed an immediate and drastic loss of weight (**Fig 8A**). However, while almost all wildtype CPXV-infected mice had to be sacrificed due to more than 20% weight loss at the dose used, most mice infected with CPXVΔ14 recovered their weight loss and survived (**Fig 8A**). To determine whether this attenuation was due to a reduction in viral replication and dissemination we sacrificed five CPXV or CPXVΔ14-infected mice at day 10 post-infection and measured copy numbers of viral genomes in the spleen by qPCR. Consistent with a reduction in viral replication we observed a significant reduction of more than 10fold in CPXVΔ14 genome copy numbers compared to CPXV (**Fig 8B**). Similarly, we observed a CXPV14-dependent increase in viral infection of ovaries when mice were infected intraperitoneally with either CPXV or CPXVΔ014 (**Fig 8B**). These data suggest that CPXV14 is required for viral replication and dissemination *in vivo* and contributes to virulence.

To determine whether the increased virulence and replication of CPXV14-expressing virus compared to the deletion mutant was a consequence of CPXV14 modulation of FcγR function, we monitored virulence in knockout mice that lacked the inhibitory receptor FcγRIIB [36]. FcγRIIB^-/-^ mice were intra-nasally infected with CPXV, CPXVΔ12 or CPXVΔ14 and weight loss and mortality was monitored. Interestingly, mice infected with CXPVΔ14 displayed weight loss and mortality rates that were similar to wildtype virus (**Fig 8C**). In contrast, CPXV lacking the TAP-inhibitor CPXV12 was still attenuated consistent with the previous demonstration that deletion of CPXV12 reduced virulence due to increased control by CD8+ T cells [9,16]. This result is consistent with CPXV14 contributing to virulence via FcγRIIB.

The inhibitory low affinity FcγRIIB receptor is the only Fc receptor expressed in B cells where it regulates antibody production [37]. As a result, FcγRIIB KO mice respond with increased antibodies to antigen challenge [36]. However, FcγRIIB can also act immunostimulatory by hyper-cross linking of antibodies [38,39]. Thus, CPXV14 could act through FcγRIIB both by competing with immunostimulation or by directly engaging this receptor thus dampening the antibody response.

To determine the impact of CPXV14 on the development of antibody responses, we infected female C57BL/6 mice i.p. with a non-lethal dose of VACV, CPXV or CPXVΔ14 and longitudinally monitored the development of CPXV specific neutralizing antibody titers. 2-fold serum dilutions were mixed with 80–100 PFU of CPXV followed by incubation (2h at 37°C) and quantitation of remaining infectious virus by plaque assay. VACV infected mice displayed on average the highest neutralizing titers, shown as fifty percent neutralization (CPXV_NT50_), whereas was neutralizing antibodies were significantly lower in from the CPXV- infected mice (**Fig 8D**). Interestingly, CPXVΔ14-infected mice had on average higher neutralizing Ab titers compared to CPXV but the titers tended to be lower than that of VACV. These results suggested that CPXV14 contributed to the reduced antibody response observed for CPXV compared to VACV.

### CPXV14 reduces CD8+ T cell responses to CPXV

**Fig 9:**
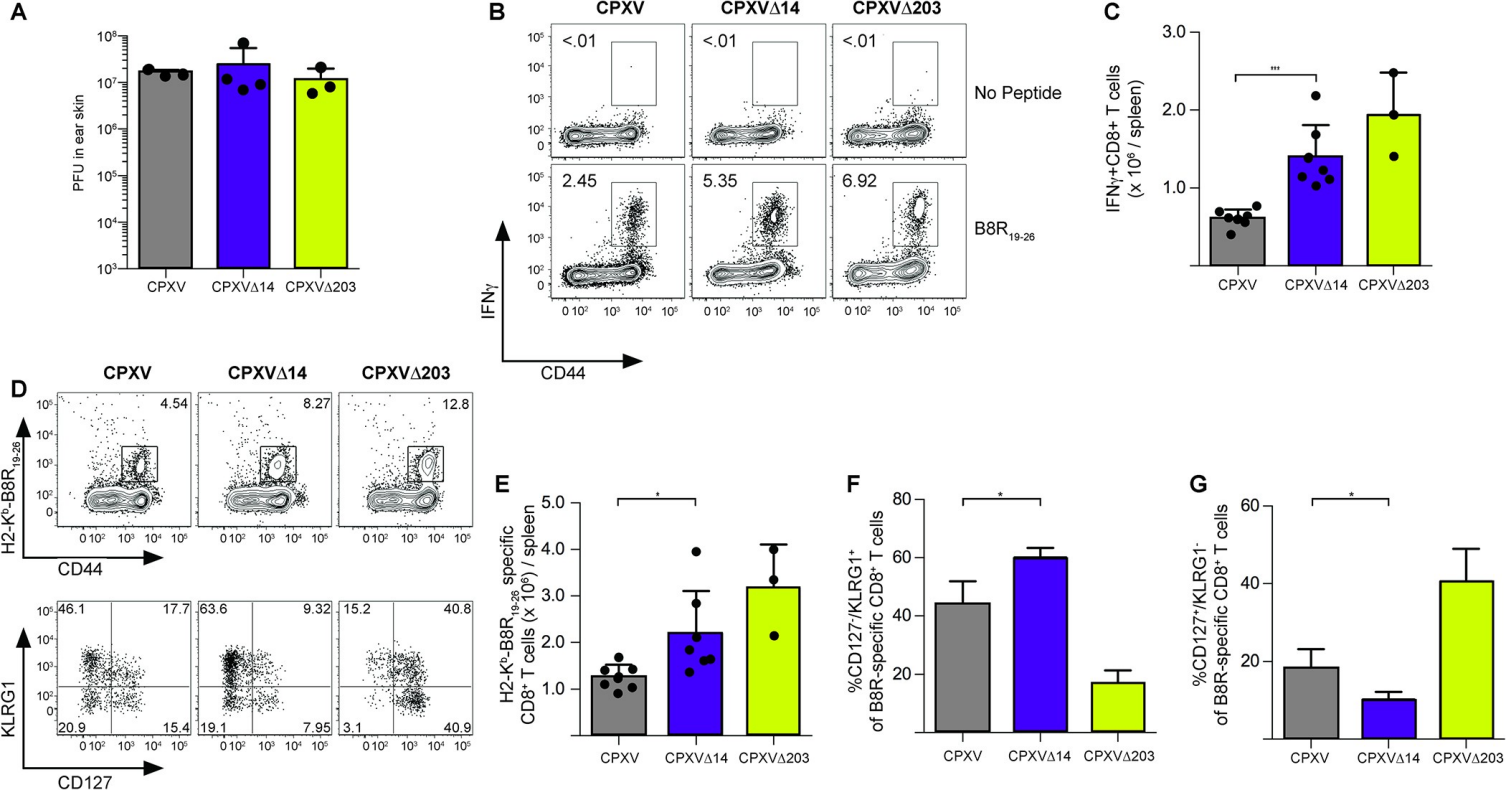
Deletion of CPXV14 increases CPXV-specific CD8+ T cell responses *in vivo*. **A)** Deletion of CPXV14 or CPXV203 does not impact local replication. The left ears of C57BL/6J B6 mice were inoculated by scarification with 1 x 10^5^ PFU of CPXV (n = 3), CPXVΔ14 (n = 4), or CPXVΔ203 (n = 3). On day 10 post-infection, viral load in the infected ear was quantified using standard plaque assay. **B)** Increased CPXV-specific T cell responses upon deletion of CPXV14 or CPXV203. Infection with the indicated viruses was performed as in (A). On day 10 post-infection, splenocytes were stimulated with B8R_19-26_ peptide for 5h and IFNγ expression was analyzed by ICS. **C)** Quantification of (B) for CPXV (n = 7), CPXVΔ14 (n = 7) or CPXVΔ203 (n = 3). **D)** T cell phenotyping of CPXV-specific T cell response. The experiment was performed as in (B) except that B8R_19-26_-specific CD8+ T cells were identified with MHC-I tetramers and expression of CD127 and KLRG1 was analyzed. **E)** Quantification of total B8R-specific CD8+ T cells from (D); CPXV (n = 7), CPXVΔ14 (n = 7) or CPXVΔ203 (n = 3). **F & G)** Quantification of CD8+ T cell phenotypes from (D) for terminally differentiated effectors (CD127-/KLRG1+) (F) or memory precursor cells (CD127+/KLRG1-) (F). For all charts shown in Fig 8 statistical significance was determined using 2-way ANOVA with Tukey’s post-test (* p≤0.05, ** p≤0.01, ** p≤0.001).

To determine whether CPXV14 also impacted the T cell response to CPXV we used the ear scarification model to monitor local and systemic T cell activation in mice infected with CPXV, CPXVΔ14 and CPXVΔ203, the latter lacking the CPXV203 protein that retains MHC-I in the ER [15]. Mice were inoculated with 1x10^5^ PFU of CPXV, CPXVΔ14 or CPXVΔ203 through scarification of the left ear skin. No difference in viral loads was observed when viral titers were measured in infected ears at ten days post-infection (**Fig 9A**) suggesting that local viral replication was not affected by CPXV14 unlike systemic replication measured above. Splenocytes were harvested at the same time point and T cell activation was determined by ICS upon stimulation with the immunodominant Kb-restricted peptide B8R_19-26_ (TSYKFESV). The average frequencies of B8R-specific CD8+ T cells increased significantly in mice inoculated with CPXVΔ14 compared to CPXV (**Fig 9B and 9C**). A similar increase was observed when T cells were stained with H2-K^b^-B8R tetramers (**Fig 9D and 9E**). Interestingly, the B8R-reactive CD8+ T cells displayed an increased terminally differentiated effector phenotype (CD127-/KLRG1+) and a decreased memory precursor cell phenotype (CD127+/KLRG1-) in CPXVΔ14-infected mice compared to CPXV-infected mice, whereas the opposite was observed for CPXVΔ203 (**Fig 9D, 9F, and 9G**). Taken together these data suggest that, in addition to affecting antibody responses, CPXV14 also dampens the T cell response to CPXV.

## Discussion

We report here that CPXV14 is a secreted viral protein that binds with high affinity to FcγRs and contributes to virulence and downregulation of antibody and T cell responses in infected mice. We discovered the function of CPXV14 by its ability to inhibit antibody-mediated activation of T cells *in vitro*. However, this inhibition seems to be an indirect effect of CPXV14 preventing antibody binding to FcγRs on bystander cells, a previously described, but often overlooked, aspect of this *in vitro* assay. Surprisingly, even plate-bound, and thus immobilized, antibodies required FcγR on bystander cells and could be inhibited by CPXV14 or by α-FcγR antibodies. Thereby we observed a differential FcγR requirement for CD8+ and CD4+ T cells in ICS versus proliferation assays with bystander FcγRs being required more for CD8+ than CD4+ T cells in ICS whereas CD4+ T cells, but less so CD8+ T cells, required bystander FcγRs for proliferation. Interestingly, it is thought that FcγRs expressed on bystander cells were responsible for the severe cytokine storm caused by α-CD28 “superagonist” antibody in clinical trials [40–43]. Our data suggest that CPXV14 or related proteins from other poxviruses might be able to prevent such unwanted side-effects of therapeutic antibody treatments.

FcγRs are involved in many aspects of innate and adaptive (both humoral and cellular) immunity to viral infections. Mice encode four whereas humans encode six FcγRs (reviewed in [44]): FcγRI (CD64) is a high affinity activating receptor expressed on tissue macrophages and monocyte-derived dendritic cells. The low affinity, inhibitory FcγRIIB (CD32B) is expressed on a wide variety of cells where it performs a number of immune regulatory functions [37]. It is the only FcγR expressed on B cells where it counteracts the activating signals of the B cell receptor, thus limiting B cell expansion. Interestingly, it was recently reported that FcγRIIB is also expressed on a sub-population of mouse CD8+ T cells where it triggers apoptosis upon binding to the immunosuppressive cytokine Fgl2 [45,46]. Thus, the reduction of CD8+ T cell responses observed in CPXV compared to CPXVΔ14 could be due to a direct inhibitory effect of CPXV14 on T cells. Humans additionally express activating versions, FcγRIIA and FcγRIIC. The affinity of mouse FcγRIV to IgG is higher than that of FcγRIIB and FcγRIII, but lower than FcγRI. Our observation that CPXV14 inhibits FcγRIIB and FcγRIII signaling in response to IgG at lower concentrations compared to FcγRIV, whereas FcγRI is not inhibited, despite binding CPXV14 (**Figs 6 and 7**), is thus in perfect correlation with the known binding affinities of these FcγRs to IgG and a competitive binding mechanism for CPXV14.

In addition to IgG, FcγRs are also known to bind host proteins of the pentraxin family such as C-reactive protein which can result in both interference with IgG binding and stimulation of FcγR signaling [47]. Similarly, we observed that CPXV14 can both interfere with IgG binding to FcγRs (except for FcγRI) but also activate FcγRs when immobilized. Although the latter was only observed when CPXV14 was immobilized *in vitro* it is conceivable that CPXV14 could be cross-linked *in vivo* by anti-CPXV14 antibodies. Moreover, the FcγR-binding C-terminal SECRET domain of the ECTV003-encoded CrmD protein naturally occurs as a fusion protein with an N-terminal TNF-receptor homologous domain that would be expected to bind to TNFα as a heterohexamer [48] thus potentially creating a FcγR-binding protein complex. Interestingly, the CTD of CPXV221 encoding the CrmD homolog did not inhibit antibody-dependent T cell stimulation despite differing by only three amino acids from ECTV003 (**Fig 3**). However, since this inhibition is predominantly mediated via FcγRIIB (**Fig 5**) these experiments do not rule out that the CTD of CPXV221 binds to other FcγRs.

The dual function of chemokine and FcγR binding of CPXV14 together with the multiple roles of FcγRs in modulating T and B cell responses renders it difficult to directly link *in vitro* observations of CPXV14 function with *in vivo* outcomes. For instance, chemokine sequestration could contribute to the reduced local T cell response observed for WT compared to CPXV14-deleted virus in the ear scarification model. Interestingly, virulence was restored in mice lacking the FcγRIIB receptor suggesting that the interaction with FcγRIIB is a key element of CPXV14’s role in virulence. While FcγRIIB has low affinity to monomeric IgG, it can engage multimeric IgG/Ag complexes with high avidity. Such receptor clustering results in inhibitory signaling through its ITIM domain which counteracts positive signals from other receptors, including positive signals from other FcγRs or the B cell receptor [49]. However, FcγRIIB also mediates the uptake of IgG/Ag complexes by Ag presenting cells which supports the induction of T cell response. Conceivably, CPXV14 could interrupt such interactions *in vivo* which would provide an alternative explanation for the decreased T cell responses observed in the ear-scarification model for CPXV14-intact virus.

To our knowledge, CPXV14 and related proteins from poxviruses are the first viral proteins shown to directly engage the host’s FcγRs. However, an indirect inhibition of FcγR-function by high levels of antigen/antibody complexes was reported in mice chronically infected with LCMV [50,51]. Moreover, bacterial pathogens such as *S*. *aureus* are known to express protein A that binds to the Fc domain of IgG thus interfering with their FcγR binding and, in addition, secrete proteases that cleave IgG [52]. Furthermore, pathogenic *E*.*coli* interferes with phagocytosis through a low avidity interaction with FcγRIII [53]. An alternative strategy for preventing Ab-mediated activation of FcγRs is the expressing of viral FcγRs as frequently found in the herpesvirus family, including herpes simplex [54]. Furthermore, cytomegalovirus encodes three different types of FcγRs whose expression on infected cells counteracts the activation of host FcγRs on effector cells by antibodies bound to infected cells [55,56].Thus, interfering with Ab/FcγR interaction by pathogens is frequently observed but a direct targeting of FcγRs seems to be a new mechanism.

Since FcγRs are key regulators of immune responses [49], the discovery of viral proteins that target these receptors might also open the possibility of using viral proteins therapeutically, a possibility that has been explored for other poxviral immune modulators [57]. In sum, we described a novel viral immune evasion strategy that is conserved in virulent poxviruses and that has the potential to be used to target an important class of immune regulatory proteins.

## Materials and methods

### Ethics statement

All experiments utilizing animals were reviewed and approved by the Oregon Health and Science University (OHSU) Institutional Animal Care and Use Committee (IACUC) protocol #0865 and the OHSU Institutional Biosafety Committee (IBC) registration number: IBC-07-07. Maintenance and all experiments involving mice complied with all IACUC and IBC regulations and associated protocols. Use of all viruses and recombinant viruses for these studies, including *in vitro* and *in vivo* experiments, were reviewed and approved by the OHSU IBC (registration number: IBC-07-07) and OHSU IACUC (protocol #0865).

### Mice

BALB/c mice, C57BL6 mice, and FcγRIIb^-/-^ (CD32b^-/-^) mice on a mixed C57BL6;129 genetic background [36] were purchased from The Jackson Laboratories (Bar Harbor, ME, USA).

### Cell lines and viruses

BSC 40 (ATCC; CRL2761), Chinese Hamster Ovary (CHO-K1) (ATCC; CCL61), and MC57G mouse fibroblast cells (ATCC; CRL2295) were maintained in DMEM (Corning) supplemented with 10% Fetal Bovine Serum (FBS) (Hyclone). A20 mouse B lymphoma cells (ATCC; TIB208) were maintained in RPMI 1640 (Corning) supplemented with 10% FBS. 293-F human embryonal kidney cells (Thermo Fisher Scientific; Cat.#: R79007) were maintained in 293 expression media (Thermo Fisher Scientific; Cat.#: 12338026).

VACV (Western Reserve) and CPXV (Brighton Red Strain; BR) recombinants were propagated in BSC40 cells maintained in MEM (Corning) supplemented with 5% FBS. Viruses were purified utilizing standard protocols, as we have previously reported [16].

BW5147 mouse thymoma cells (kindly provided by Ofer Mandelboim, Hadassah Hospital, Jerusalem, Israel) were maintained at 3x10^5^ to 9x10^5^ cells/ml in RPMI GlutaMAX (Gibco) supplemented with 10% (vol/vol) FCS, sodium pyruvate (1x, Gibco) and β-mercaptoethanol (0.1 mM, Sigma). Generation of BW5147 reporter cells stably expressing chimeric mouse FcγRs is described elsewhere [58].

### *In vitro* naïve T lymphocyte activation assay

Mouse T lymphocyte activation: antibodies hamster α-mouse(m) CD3ε (Clone: 145-2C11; BD Biosciences (BD); Cat.#: 553057) and hamster α-mCD28 (Clone: 37.51; BD; Cat.#: 553294) were coated (both at 10μg/ml) in sterile PBS overnight (18h) on 96 well flat-bottom plates (Fisher Scientific) at 4°C. A20 mouse B lymphoma cells were infected with viruses at a multiplicity of infection (MOI) of 5 overnight (18h). Naïve, specific pathogen free (SPF) BALB/c mice (age 9–12 weeks and sex matched) were euthanized and spleens were harvested. Red blood cells (RBC) were lysed with Ammonium-Chloride-Potassium (ACK) lysis buffer (Lonza) and then centrifuged (3,000 rpm, 5min). Splenocytes were isolated and resuspended in RPMI 1640 supplemented with 10% FBS (Hyclone-Fisher Scientific). Infected A20 cells were washed thoroughly three times to remove residual virus and resuspended in RPMI 1640 supplemented with 10% FBS. Infected A20 cells were stained with an anti-OPXV antibody, specific for OPXV virion proteins to confirm infection, as previously described [14]. Splenocytes and infected A20 cells were mixed at a 2:1 ratio (splenocyte:A20) and incubated at 37°C for 4h. Antibody (α-mCD3ε and α-mCD28) coated plates were thoroughly washed three times with sterile phosphate buffered saline (PBS). Next, Brefeldin A (BFA) (MilliPORE Sigma; Cat.#:B6542) was added to the combined splenocyte:A20 cell mixture and transferred onto the antibody coated plates. Plates were centrifuged (1,200 rpm, 1min) and incubated at 37°C for 6h. Cells were then washed and surface stained with the following antibodies: hamster α-mCD3ε PerCp5.5 (Clone: 145-2C11; eBiosience; Cat. #: 45-0031-82), rat α-mCD4 Pacific Blue (Clone: RM 4–5; BD; Cat.# 558107), rat α-mCD8α R-phycoerythrin (PE)-Cy7 (Clone: 53–6.7; eBiosience; Cat#:25-0081-82), Live/Dead Fixable Aqua Dead Cell Stain kit (0.5μl) (Invitrogen; Cat.#:L34957) in the presence of “Fc Block” (0.83μl) [rat α-mCD16/CD32] (Clone: 2.4G2; BD; Cat.#: 553142) and IgG antibodies from mouse serum (MilliPORE SigMGA; Cat.#: I8765) overnight (16h) at 4°C. Next, the cells were subjected to Intracellular Cytokine Staining (ICS). Briefly, cells were thoroughly washed three times and fixed and permeabilized with CytoFix/Cytoperm kit (BD). Following thorough washing, rat-anti-mouse IFNγ-FITC (1μl) (Clone: XMG1.2; BD; Cat.#: 554411) and rat-anti-mouse TNFα-PE (1μl) (Clone: MP6-XT22; Biolegend; Cat.#: 506306) antibodies were added and incubated at 4°C for 1h. Cells were washed three times and data was collected on a LSRII flow cytometer with FACSDiva software, both manufactured by BD. Data was analyzed with FlowJo software (BD). Singlet, live, CD3ε^+^ T lymphocytes were gated on either CD4^+^ or CD8α^+^ subpopulations as depicted in the flow cytometry gating scheme illustration (**S1 Fig**). Single color antibody staining controls were utilized to establish and verify the staining panel accuracy.

For supernatant experiments, MC57G cells were infected with indicated viruses at an MOI = 2 for 18h. Supernatants were harvested and virus was removed by ultracentrifugation (18,000 rpm, 80 min, 4°C). SPF BALB/c mouse (6 weeks old, sex matched) splenocytes were pre-treated for 2h with a 50:50 ratio of supernatants to RPMI 1640 supplemented with 10% FBS (supernatant:media) prior to stimulation with plate bound α-CD3 and α-CD28 in the presence of BFA for 6h. Infected MC57 cells were stained with an anti-OPXV virus antibody, as described above. Cells were surface stained followed by ICS, as described above. Data was collected on a LSRII and then analyzed with FlowJo software as described above.

For CPXV14-His recombinant protein experiments, splenocytes were treated with indicated amounts of protein for 4 hours prior to antibody stimulation and allowed to remain in the culture throughout the stimulation.

### Generation of recombinant viruses

CPXV mutants A694, A518, A530, A531, A529, and A624 were generously provided by David Pickup, Duke University.

CPXV Δ204–221 (A694) has been described previously [59]. The genome of this variant has lost the 33.7 kb region from nucleotide 190,832 to the right-hand end of the genome (nucleotide 224,499), with the deleted region replaced by an inverted copy of the left-hand end of the genome encompassing nucleotides 1–15,461 [19].

CPXV Δ11–38 (A518): The CPXV (BR) A518 recombinant virus disrupted or deleted ORFs CPXV11-38 and has been described previously by [16].

CPXVΔ11–16 (A530): A 5430 contains a deletion of nucleotides 10642–17410 in the CPXV-BR genome, disrupting or deleting the 6 ORFs CPXV011-016 as described in [60]. Plasmid p1538 DNA was cut with ClaI, and the vector-containing fragment (with terminal ClaI sites corresponding to those at nucleotides 10642 and 17410) was re-circularized by ligation to generate plasmid p1655. The 2.0-kbp EcoRI fragment from plasmid pTK61-gpt [61], containing the *E*. *coli gpt* gene under the control of the promoter of the vaccinia virus 7.5K ORF, was inserted into the filled-in ClaI site of plasmid p1655 to create plasmid p1659. This plasmid was used to construct a recombinant CPXV containing the replacement of nucleotides 10642–17410 by a functional copy of the *gpt* gene according to the method of [61] as described above.

CPXVΔ16–27 (A531): A531 contains a deletion of nucleotides 16366–30381 in the CPXV-BR genome, disrupting or deleting 12 ORFs CPXV16-27 as described previously [60]. A 4.5 kb EcoRI-ClaI fragment (nucleotides 11912–16366 of CPXV-BR) of p1538 DNA was inserted into the EcoRI-ClaI sites in pGEM7zf(+) to generate plasmid p1649. The 120 bp ClaI-BamHI fragment (nucleotides 30381–30500 CPXV-BR) was inserted into the ClaI-BamHI sites (nucleotides 4454 and 4465) of p1649 to create plasmid p1653. This plasmid was used to construct a recombinant CPXV containing the replacement of nucleotides 16366–30381 by a functional copy of the *gpt* gene according to the method of [61] as described above.

CPXVΔ25–38 (A529): A529 contains a deletion of nucleotides 28099–39152 in the CPXV-BR genome, disrupting or deleting the 14 ORFs CPXV25-38 as described previously [60]. The 9.9kb PstI-HindIII fragment (nucleotides 9337–19300) of the PstI F fragment of CPXV-BR DNA was cloned into HindIII-PstI cut pUC19 vector to generate plasmid p1538. The 0.9kb PstI-ClaI fragment (nucleotides 25284–28099 of CPXV-BR DNA was ligated to the 4kb ClaI–HindIII fragment of p1538 (containing nucleotides 9337–10642 of CPXV-BR DNA) to generate plasmid p1595. The 2.6 kb PstI- ClaI fragment (nucleotides 25284–28099 of CPXV-BR DNA) was ligated to the 3.6 kb PstI-ClaI fragment of p1595 to generate plasmid p1648. The 2.0-kbp EcoRI fragment from plasmid pTK61-gpt [61], containing the *E*. *coli* xanthine-guanine phosphoribosyltransferase (*gpt*) gene under the control of the promoter of the vaccinia virus 7.5K ORF, was inserted into the filled-in ClaI site of plasmid p1648 to generate plasmid p1657. This plasmid was used to construct a recombinant cowpox virus containing the replacement of nucleotides 28099–39152 by a functional copy of the *gpt* gene according to the method of [61]. The extent of the deletion in the DNA of the recombinant virus was verified by DNA hybridization analyses of DNA extracted from cells infected with the recombinant virus.

CPXVΔ15 (A624): The recombinant CPXVΔ15 (A624) with an inactivated CPXV15 (*vCD30*) gene was constructed as previously reported [24].

CPXVΔ14 was generated through *in vivo* homologous recombination as we have previously described with the generation of CPXVΔ12 [17], utilizing previously described protocols [62,63]. Plasmid pCR2.1_Δ14CPXV was constructed by splicing 150 base pair (bp) upstream and downstream regions of CPXV14 to the 5’ and 3’ terminal ends of the GFP-GPT cassette, respectively, utilizing the Splice Overlap Extension Polymerase Chain Reaction (SOE–PCR) method [64]. The 3’ and 5’ terminal ends of CPXV14 were cloned by PCR amplification of genomic CPXV-BR utilizing the following primers: d14Int#1 (5’-AACACAATACTAATATTCGCATCATTATTTGTTGCATC-3’), d14Int#2 (5’-GCTATTTTTAAATCCATATGACTAGTAGATCCTCTAGAACAAGATGATACGTTAACTGGATATAGTTCAAAATCTATAG-3’), d14Int#5 (5’-GCAAACCTGCGGATCCGCTCTAGAGAAAATTTCACTTTAATAGGCAACTGTCTATCAGATC-3’), d014Int#6 (5’-AGCGCACATATTTGGAGGTATACCGTTAATATC-3’), respectively. The pT7 E/L EGFP-GPT Vector [65] was kindly provided by Dr. Grant McFadden (Arizona State University; Tempe, AZ, USA) and cloned from the following primers: d14Int#3 (5’-CTATAGATTTTGAACTATATCCAGTTAACGTATCATCTTGTTCTAGAGGATCTACTAGTCATATGGATTTAAAAATAGC-3’) and d014Int#4 (5’-GATCTGATAGACAGTTGCCTATTAAAGTGAAATTTTCTCTAGAGCGGATCCGCAGGTTTGC-3’). The EGFP-GPT cassette, the 3’ and 5’ termini of CPXV14 segments were then fused together by SOE–PCR using primer pair d014Int#1 and d014Int#6. The construct was then cloned into pCR2.1 TOPO-TA vector (Invitrogen) resulting plasmid, pCR2.1_ΔCPXV14, was propagated in *OneShot Top* 10 chemically competent *E*. *coli* (Thermo Fisher, Cat.#: 404010). pCR2.1_ΔCPXV14 was transfected into 1x10^6^ BSC40 cells with Lipofectamine 2000 transfection reagent (ThermoFisher). Three hours post-transfection cells were infected with CPXV-BR (MOI = 1) and were cultured under GPT selection media using 250 μg/ml xanthine, 15 μg/ml hypoxanthine, and 32 μg/ml mycophenolic acid (MilliPORE Sigma; Cat.# X0626, H9377 and M5255, respectively). The resulting EGFP-positive virus was isolated using three successive rounds of plaque purification under the same selection media. PCR amplification of CPXV14 with primers L-152-F (5’-AGAAGCTGTACGAGCATAGTAACTTTTTATCAGACG-3’) and R-188-R (5’-ACAATCATGTGGACCGGATAAACCACGA-3’) from genomic DNA from CPXV-BR and CPXVΔ14 viruses confirmed the deletion of the CPXV14 ORF in CPXVΔ14. The presence of the EGFP cassette in CPXVΔ14 was confirmed by PCR.

Next-generation-sequencing (NSG) on a Illumina Miseq sequencer, with Geneious 8.1.4 software for analysis, confirmed no errors or unintended mutations in the protein coding sequence of the CPXVΔ14 recombinant virus compared to the wildtype CPXV (Brighton Red Strain; Genbank accession #: AF482758).

CPXVΔ12 was generated previously as described [17].

### Lethal challenge

Lethal challenge studies were conducted by intra-nasal (IN) infection following brief (<1 min) anesthesia with isoflurane (Piramal Critical Care; Bethlehem, PA; USA) using either 200,000 plaque-forming units (PFU) or 900,000 PFU of indicated virus in a 20μl volume of sterile PBS for wildtype (C57BL/6) mice or (FcγRIIb^-/-^[CD32b^-/-^] knockout mice on a C57BL/6 background, respectively.

### Quantification of CPXV and T Cell responses from infected skin

Quantification of viral load in the infected skin was determined using standard plaque assays on BSC-40 cells. Briefly, infected ears were removed and homogenized in 1 ml of RPMI supplemented with 1% fetal bovine serum. Skin homogenates were then subjected to three rounds of freeze-thaw before serial dilutions were inoculated on BSC-40 cells in a 12-well plate that were then covered with 1% Seakem agarose in Modified Eagle Medium (Gibco). Plaques were visualized three days later following overnight incubation with Neutral Red dye.

Infections with CPXV were performed on anesthetized mice by placing 1 x 10^5^ PFU of virus in 10 μl of PBS on the ventral side of the ear pinna and then poking the virus coated skin 25 times with a 27G needle.

For detection of IFNγ, splenocytes were incubated for 5h with 500 nM of B8R_19-26_ (TSYKFESV) peptide (Bio-synthesis) in the presence of BFA (BioLegend). ICS was performed using the CytoFix/CytoPerm kit (BD Biosciences) according to the manufacturers protocol. Briefly, following the staining of surface antigens cells were incubated with 100 μl of Cytofix/Cytoperm for 10 min at 4°C and washed once with Perm/Wash buffer. Staining for IFNγ was performed in Perm/Wash buffer for 20 min at 4°C.

H2-K^b^-B8R_19-26_ (TSYKFESV) tetramers (National Institute of Health Tetramer Core Facility). Staining for surface antigens was performed in PBS/1% fetal bovine serum for 15 min at 4°C. For tetramer binding, cells were incubated for 45 min at room temperature. Data was acquired using either a BD Fortessa or BD LSRII Flow Cytometer in the OHSU Flow Cytometry Core Facility. Antibody clones: CD127 (A7R34, BioLegend), KLRG1 (2F1, Tonbo).

### Determining CPXV viral titers in ovaries

Female, naïve, SPF C57BL/6 mice (11 week old) were infected intraperitoneally (IP) with 2x10^6^ PFU of indicated virus in PBS with a total volume 100μl. Mice were euthanized and ovaries were harvested on days 5, 6 and 10 post-infection. Ovaries were frozen at -80°C. Once thawed, ovaries were rigorously homogenized in a 240μl volume of sterile DMEM media with no supplements and refrozen at -80°C. Homogenates were thawed and serial diluted in DMEM media supplemented with 5% FBS and titered on Vero cells.

### CPXV genome copy number quantification

Quantitative PCR (qPCR) was employed to determine CPXV genome copy numbers as previously described [16]. Briefly, forward primer (5’-CGGCTAAGAGTTGCACATCCA -3’) and reverse primer (5’-TCTGCTCCATTTAGTACCGATTCTAG-3’) hybridized at positions 2048–2069 and 2091–2118, respectively, and a probe (5’- (6FAM)-AAGATCATTCTACGT-(MGB)-3’) were used for the qPCR reactions. A positive control plasmid was generated by cloning the same target sequence (positions 2048–2118) into a mammalian expression vector plasmid (pUHD10.1) with a forward primer (5’- CCGCGGCGGCTAAGAGTTGCACATCCA- 3’) and reverse primer (5’-CCCGGGTCTGCTCCATTTAGTACCGATTCTAG-3’) into the *Xma*I and *Sac*II sites of pUHD10.1. The standard curve for each qPCR reaction plate (96 wells) was generated by 10-fold serial dilution of the pUHD10.1 positive control plasmid. Naïve, SPF C57BL/6 mice (age and sex matched) were infected IN with 200,000 PFU of indicated virus after a brief (<1 min) anesthesia with isoflurane. Ten days post infection, spleens were harvested, individually weighed and homogenized in MEM (Corning), supplemented with 2% FBS, and frozen at -80°C. Genomic DNA was purified with DNeasy Blood & Tissue Kit (Qiagen), following thawing.

### CPXV14 His-tag protein plasmid construction, purification and characterization

CPXV14-His protein was expressed from pFM1-2R_SCP-2_CPXV_BR plasmid by transfection of HEK293F cells and purified from the cell culture supernatants. 200ml shaker cells cultures of 293F cells at 1 x 10^6^ cells/ml were transiently transfected with 200μg of the pFN1-2R_SCP-2_CPXV-BR plasmid using linear polyethylene (Polysciences Inc., Cat.#: 23966). Supernatants were harvested three and six days later and stored at -80°C. CPXV14-His protein was purified by passing supernatants over a NiNTA Superlow resin (Qiagen; Cat.#:30410). Eluted protein was then dialyzed twice in PBS and then concentrated utilizing an Ultracel 10K columns (Amicon; Ultra-15). CPXV14-His protein was aliquoted, flash frozen and stored at -80°C. N-Linked glycosylation release was performed by incubating purified CPXV14-His protein overnight (18h, 37°C) with PNGase F (MilliPORE Sigma) or buffer only negative control.

The 6-Ala mutant of CPXV14-His was generated by replacing the charged residues D43, E45, E169, E170, E172, E173 with Alanines. The mutant sequence was custom synthesized by GeneWiz and substituted for the original sequence in CPXV 14-His by replacing a SalI and Bsu36I restriction fragment. In the negative recombinant control proteins ECTV003-3Lys and ECTV003-3Ala the residues D167, E169, D316 in ECTV003-His were replaced with Lysines or Alanines, respectively [66]. The mutant proteins were generated, produced and purified as described above.

### Biolayer Interferometry Binding Analysis

The apparent binding affinity of CPXV14 was measured by BLI using an Octet-Red96 device (Pall ForteBio) as described previously [67]. Recombinant murine FcγRIIB proteins were obtained from R&D systems (R&D 1460-CD). Biotin-labeled CPXV14 was loaded onto streptavidin biosensors until saturation, typically 10 μg/mL for 2 min in PBS (pH 7.4) and 0.005% (vol/vol) Tween-20. Association and dissociation were measured at 25°C. The real-time data were analyzed using Biaevaluation 3.1. Steady-state equilibrium concentration curves were fitted using a 1:1 binding model to obtain an apparent affinity.

### Neutralizing antibody titers (NT50’s)

Naïve, SPF C57BL/6 mice (11 week old, female) were infected IP with 1x10^6^ P of indicated virus in PBS with a total volume 100μl. Mice were euthanized and blood was collected 14, 28 or 61 days post-infection. Once blood was fully coagulated (4h at room temperature), blood was centrifuged at 13,300 rpm for 1 minute. Serum was collected and frozen at -80°C.

Neutralizing serum titers were determined by mixing 2 fold dilutions of serum with 80–100 PFU CPXV, incubating 2h at 37°C and determining remaining infectious virus by plaque assay. Data was analyzed by plotting remaining % infectious virus against the logarithm of the dilution, the dilution for fifty percent neutralization was then determined by linear interpolation.

### FcγR binding and activation assays

FcγR activation assessment was performed as described earlier [34,68]. Briefly, antibodies, immune complexes or recombinant CPXV14 proteins were immobilized on 96well ELISA microtiter plates (NUNC maxisorp). BW5147 reporter cells were added and incubated for 16h at an E:T ratio of 20:1 at 37°C in a 5% CO2 atmosphere. Addition of titrated amounts of recombinant CPXV14 was performed concomitantly with the addition of reporter cells. Reporter cell mIL-2 secretion was quantified by subsequent anti IL-2 sandwich ELISA. TNFα immune complexes were generated by preincubation of 12 nM recombinant human TNFα (NEB) with 6 nM mouse anti-human TNFα IgG1 (R&D Systems) in PBS for 30 min at 4°C followed by immobilization for 1h at 4°C. To detect CPXV14 binding to reporter cells, BW5147 reporter cells were incubated with CPXV14 or its 6Ala mutant at 25ng/μl for 1h at 4°C. Binding was detected via flow cytometry using an anti-His-PE mAb (Miltenyi Biotec) at 1:100 dilution.

## Supporting information

S1 Fig Flow Cytometry and IntraCellular Cytokine (ICCS) Gating Strategy. Splenocytes originating from either BALB/c or C57BL/6 mice were stimulated ex-vivo with either αCD3ε and αCD28 plate bound antibodies prior to flow cytometry. Extracellular and intracellular antibody staining was analyzed using the depicted flow chart. The percentage frequencies of TNFα-expressing T cells were determined by gating on either singlet, live, small lymphocytes, CD8α+ (Dot plot label A; bottom row) or singlet, live, small lymphocytes, CD4+ (Dot plot label B; bottom row). C) depicts gating for CD44+ CD8+ T cells.
(DOCX)

S2 Fig Generation and Characterization of CPXVΔ14. A) Schematic of CPXVΔ14 construction by homologous recombination. The CPXV14 ORF was replaced with an expression cassette for enhanced green fluorescent protein (EGFP) under control of an early late promoter and the selectable *E*. *coli* Xanthine phosphoribosyltransferase (GPT) gene under control of the 7.5K promoter. B) Polymerase chain reaction (PCR) with primers flanking CPXV14 confirms replacement of CPXV14 with the GFP-GPT cassette. Genomic DNA from CPXV and CPXVΔ14 was subjected to PCR analysis using primer pair L-152-F (5’-AGAAGCTGTACGAGCATAGTAACTTTTTATCAGACG-3’) and R-188-R (5’-ACAATCATGTGGACCGGATAAACCACGA-3’). The DNA fragments were predicted to contain 941 base pairs (bp) and 2,536 bp for wild-type CPXV and CPXVΔ14, respectively. C) Next generation sequencing analysis of CPXVΔ14. Genomic DNA isolated from CPXVΔ14 was sequenced on a MiSeq sequencer (Illumina). The resulting DNA reads were aligned to the published genome sequence of CPXV-BR (GenBank accession # NC_003663) using Geneious v8.1.4 software. Sequences were identical outside the CPXV14 ORF.
(DOCX)

S3 Fig CPXV14 is a late gene that is non-essential for growth *in vitro*. A) Expression kinetics of CPXV14. BSC-40 cells were infected at MOI = 3 in the presence or absence of the late-gene inhibitor arabinose-c (40 μg/ml). RNA was isolated at the indicated timepoints and RT-qPCR was used to quantitate the amount of CPXV14, CPXV66 (a late transcript) and CPXV21 (an early transcript). Expression levels are shown relative to the cellular gene GAPDH. Data was collected using an Applied Biosciences StepOne cycler and software. Shown are average results from three individual experiments with each experiment performed in triplicate (+/- SD). B) Single step growth curves of CPXV, CPXVΔ14 and CPXVΔ12, as graphed above. BSC40 cells were infected (MOI = 2) with indicated viruses and infected cells were harvested at indicated times. Genomic DNA was isolated the CPXV genome copy numbers was determined by qPCR using the following primers: forward primer (5’-CGGCTAAGAGTTGCACATCCA -3’) and reverse primer (5’-TCTGCTCCATTTAGTACCGATTCTAG-3’) hybridized at positions 2048–2069 and 2091–2118, respectively, and a probe (5’- (6FAM)-AAGATCATTCTACGT-(MGB)-3’). For positive control, the same target sequence (positions 2048–2118) was cloned into into vector pUHD10.1 [69] with a forward primer (5’- CCGCGGCGGCTAAGAGT-TGCACATCCA- 3’) and reverse primer (5’-CCCGGGTCTGCTCCATTTAGTACCGATTCTAG-3’) the XmaI and SacII sites of pUHD10.1. The standard curve for each qPCR reaction plate (96 wells) was generated by 10-fold serial dilution of the pUHD10.1 positive control plasmid. The mean of triplicate measurements is shown.
(DOCX)

S4 Fig CPXV does not inhibit T cell activation by PMA/Ionomycin or peptide. A) CPXV14 does not inhibit T cell activation by PMA/Ionomycin. Splenocytes from five female 6–10 week old BALB/cByJ mice were coincubated with virus-free supernatants from MC57 fibroblasts infected with the indicated viruses (1:1 ratio of MC57 and splenocyte SN) overnight (16 hours) at 37°C. The cells were treated with 50 ng/ml PMA, 1 μg/ml ionomycin and 4 μg/ml Brefeldin A for 6 hours at 37°C. Following stimulation the cells were washed, then stained for surface markers, followed by fixation, permeabilization and ICS for TNFα and IFNγ as shown in S1 Fig. Error bars indicate SEM. B) CPXV14 does not inhibit stimulation OT-1 T cells by SIINFEKL peptide. Splenocytes from OT-I mice (C57BL/6J background) were pre-treated with 5 μg/ml CPXV14-His (226 nM) for 4 hours and then stimulated with SIINFEKL peptide at the indicated concentrations in the presence of BFA for 6 hours. Splenocytes were surface stained and then subjected to ICS to quantitate production of OT-I specific TNFα. Plots in B show data from one representative mouse. C) Average frequency of TNFα-positive T cells of 3 mice (+SEM). The gating strategy was Lymphoctes (FSC/SSC)-> single cells -> live cells -> CD3+ -> CD8+TNFα+.
(DOCX)
