# Supplementary material for: The secreted protein Cowpox Virus 14 contributes to viral virulence and immune evasion by engaging Fc-gamma-receptors
Source: PLoS Pathog. 2022 Sep 19;18(9):e1010783. doi: 10.1371/journal.ppat.1010783 (PMC9521928; doi:10.1371/journal.ppat.1010783)
Supplement: S1 Fig — Splenocytes originating from either BALB/c or C57BL/6 mice were stimulated ex-vivo with either αCD3ε and αCD28 plate bound antibodies prior to flow cytometry. Extracellular and intracellular antibody staining was analyzed using the depicted flow chart. The percentage frequencies of TNFα-expressing T cells were determined by gating on either singlet, live, small lymphocytes, CD8α+ (Dot plot label A; bottom row) or singlet, live, small lymphocytes, CD4+ (Dot plot label B; bottom row). C) depicts gating for CD44+ CD8+ T cells. (DOCX) [file ppat.1010783.s001.docx]

**
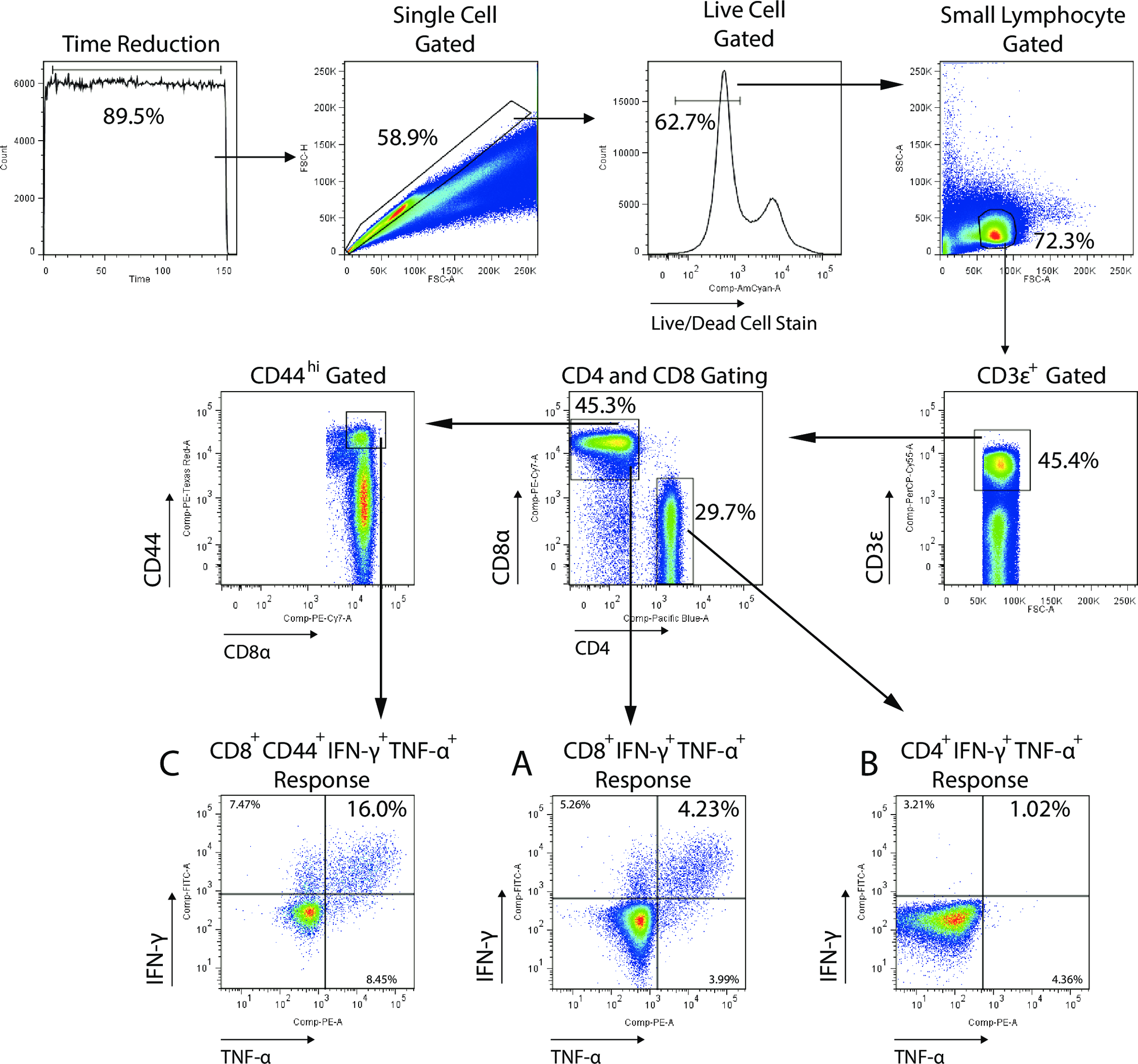
**

**S1 Fig. Flow Cytometry and IntraCellular Cytokine (ICCS) Gating Strategy.**

Splenocytes originating from either BALB/c or C57BL/6 mice were stimulated ex-vivo with either αCD3ε and αCD28 plate bound antibodies prior to flow cytometry. Extracellular and intracellular antibody staining was analyzed using the depicted flow chart. The percentage frequencies of TNFα-expressing T cells were determined by gating on either singlet, live, small lymphocytes, CD8α+ (Dot plot label A; bottom row) or singlet, live, small lymphocytes, CD4+ (Dot plot label B; bottom row). C) depicts gating for CD44+ CD8+ T cells.
