# Supplementary material for: The secreted protein Cowpox Virus 14 contributes to viral virulence and immune evasion by engaging Fc-gamma-receptors
Source: PLoS Pathog. 2022 Sep 19;18(9):e1010783. doi: 10.1371/journal.ppat.1010783 (PMC9521928; doi:10.1371/journal.ppat.1010783)
Supplement: S2 Fig — A) Schematic of CPXVΔ14 construction by homologous recombination. The CPXV14 ORF was replaced with an expression cassette for enhanced green fluorescent protein (EGFP) under control of an early late promoter and the selectable E. coli Xanthine phosphoribosyltransferase (GPT) gene under control of the 7.5K promoter. B) Polymerase chain reaction (PCR) with primers flanking CPXV14 confirms replacement of CPXV14 with the GFP-GPT cassette. Genomic DNA from CPXV and CPXVΔ14 was subjected to PCR analysis using primer pair L-152-F (5’-AGAAGCTGTACGAGCATAGTAACTTTTTATCAGACG-3’) and R-188-R (5’-ACAATCATGTGGACCGGATAAACCACGA-3’). The DNA fragments were predicted to contain 941 base pairs (bp) and 2,536 bp for wild-type CPXV and CPXVΔ14, respectively. C) Next generation sequencing analysis of CPXVΔ14. Genomic DNA isolated from CPXVΔ14 was sequenced on a MiSeq sequencer (Illumina). The resulting DNA reads were aligned to the published genome sequence of CPXV-BR (GenBank accession # NC_003663) using Geneious v8.1.4 software. Sequences were identical outside the CPXV14 ORF. (DOCX) [file ppat.1010783.s002.docx]

**
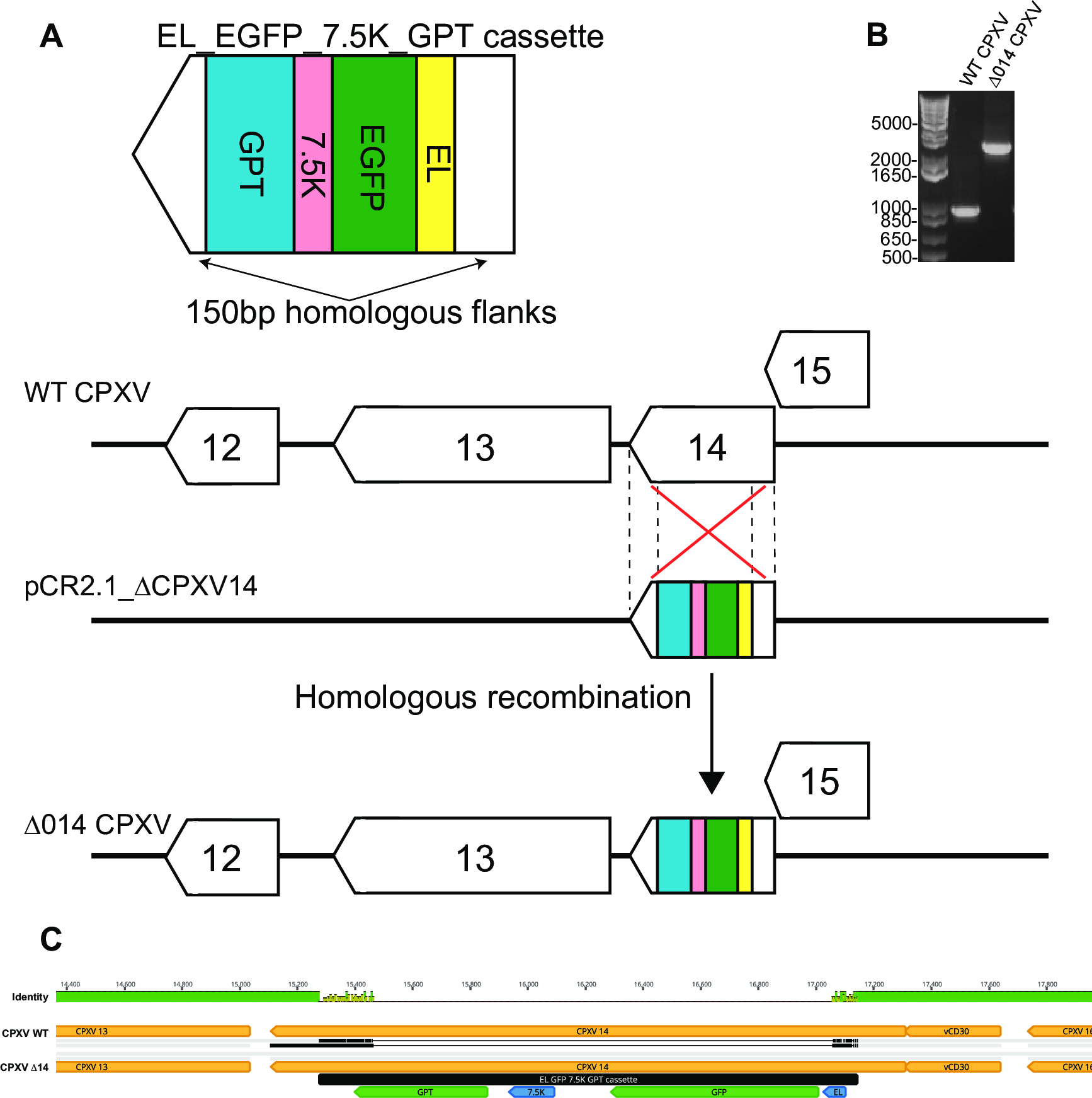
**

**S2 Fig. Generation and Characterization of CPXVΔ14**

A) Schematic of CPXVΔ14 construction by homologous recombination. The CPXV14 ORF was replaced with an expression cassette for enhanced green fluorescent protein (EGFP) under control of an early late promoter and the selectable *E. coli* Xanthine phosphoribosyltransferase (GPT) gene under control of the 7.5K promoter. B) Polymerase chain reaction (PCR) with primers flanking CPXV14 confirms replacement of CPXV14 with the GFP-GPT cassette. Genomic DNA from CPXV and CPXVΔ14 was subjected to PCR analysis using primer pair L-152-F (5’-AGAAGCTGTACGAGCATAGTAACTTTTTATCAGACG-3’) and R-188-R (5’-ACAATCATGTGGACCGGATAAACCACGA-3’). The DNA fragments were predicted to contain 941 base pairs (bp) and 2,536 bp for wild-type CPXV and CPXVΔ14, respectively. C) Next generation sequencing analysis of CPXVΔ14. Genomic DNA isolated from CPXVΔ14 was sequenced on a MiSeq sequencer (Illumina). The resulting DNA reads were aligned to the published genome sequence of CPXV-BR (GenBank accession # NC_003663) using Geneious v8.1.4 software. Sequences were identical outside the CPXV14 ORF.
