# Supplementary material for: The secreted protein Cowpox Virus 14 contributes to viral virulence and immune evasion by engaging Fc-gamma-receptors
Source: PLoS Pathog. 2022 Sep 19;18(9):e1010783. doi: 10.1371/journal.ppat.1010783 (PMC9521928; doi:10.1371/journal.ppat.1010783)
Supplement: S3 Fig — A) Expression kinetics of CPXV14. BSC-40 cells were infected at MOI = 3 in the presence or absence of the late-gene inhibitor arabinose-c (40 μg/ml). RNA was isolated at the indicated timepoints and RT-qPCR was used to quantitate the amount of CPXV14, CPXV66 (a late transcript) and CPXV21 (an early transcript). Expression levels are shown relative to the cellular gene GAPDH. Data was collected using an Applied Biosciences StepOne cycler and software. Shown are average results from three individual experiments with each experiment performed in triplicate (+/- SD). B) Single step growth curves of CPXV, CPXVΔ14 and CPXVΔ12, as graphed above. BSC40 cells were infected (MOI = 2) with indicated viruses and infected cells were harvested at indicated times. Genomic DNA was isolated the CPXV genome copy numbers was determined by qPCR using the following primers: forward primer (5’-CGGCTAAGAGTTGCACATCCA -3’) and reverse primer (5’-TCTGCTCCATTTAGTACCGATTCTAG-3’) hybridized at positions 2048–2069 and 2091–2118, respectively, and a probe (5’- (6FAM)-AAGATCATTCTACGT-(MGB)-3’). For positive control, the same target sequence (positions 2048–2118) was cloned into into vector pUHD10.1 [69] with a forward primer (5’- CCGCGGCGGCTAAGAGT-TGCACATCCA- 3’) and reverse primer (5’-CCCGGGTCTGCTCCATTTAGTACCGATTCTAG-3’) the XmaI and SacII sites of pUHD10.1. The standard curve for each qPCR reaction plate (96 wells) was generated by 10-fold serial dilution of the pUHD10.1 positive control plasmid. The mean of triplicate measurements is shown. (DOCX) [file ppat.1010783.s003.docx]

**
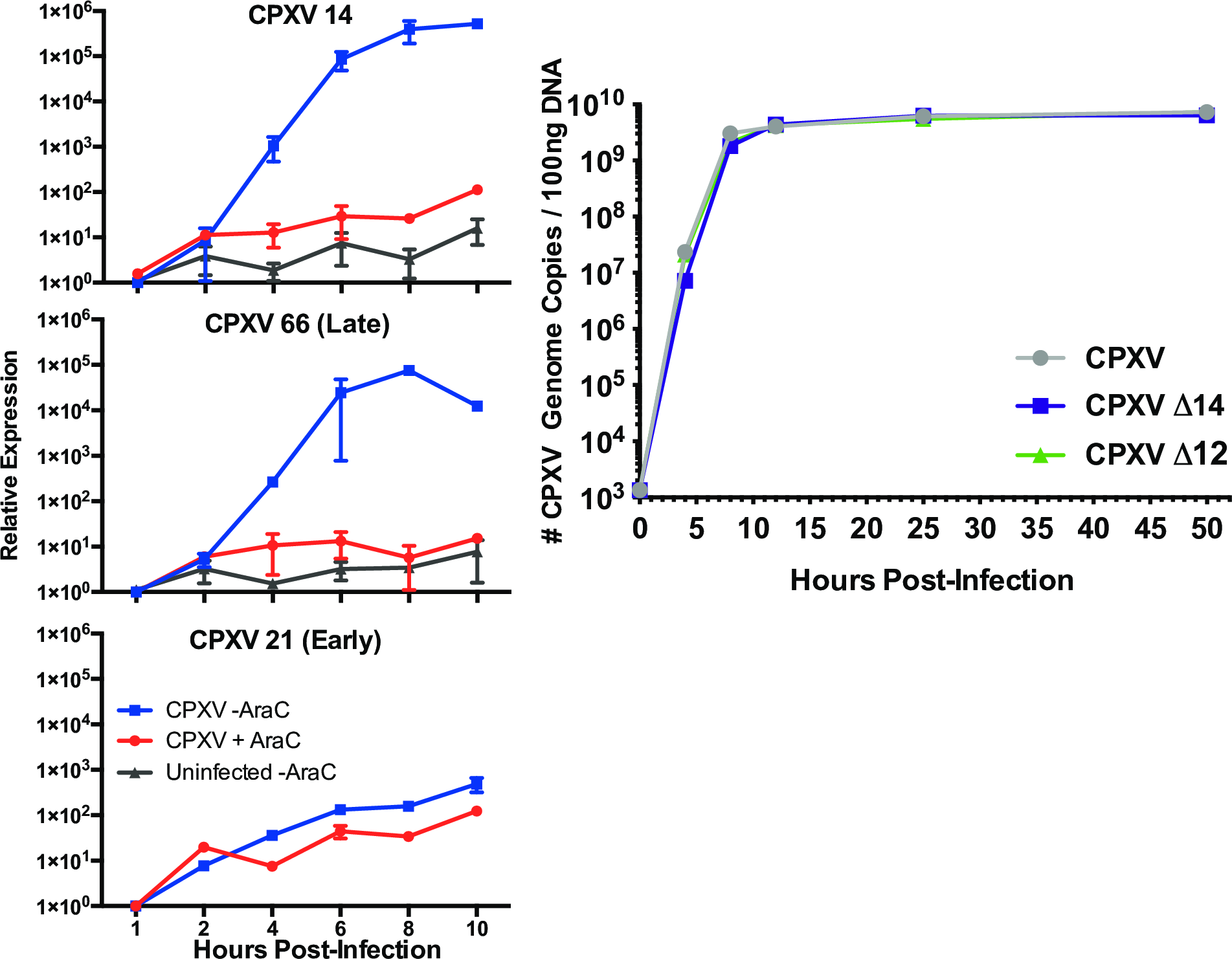
**

**S3 Fig. CPXV14 is a late gene that is non-essential for growth *in vitro.***

A) Expression kinetics of CPXV14. BSC-40 cells were infected at MOI=3 in the presence or absence of the late-gene inhibitor arabinose-c (40 μg/ml). RNA was isolated at the indicated timepoints and RT-qPCR was used to quantitate the amount of CPXV14, CPXV66 (a late transcript) and CPXV21 (an early transcript). Expression levels are shown relative to the cellular gene GAPDH. Data was collected using an Applied Biosciences StepOne cycler and software. Shown are average results from three individual experiments with each experiment performed in triplicate (+/- SD). B) Single step growth curves of CPXV, CPXVΔ14 and CPXVΔ12, as graphed above. BSC40 cells were infected (MOI = 2) with indicated viruses and infected cells were harvested at indicated times. Genomic DNA was isolated the CPXV genome copy numbers was determined by qPCR using the following primers: forward primer (5’-CGGCTAAGAGTTGCACATCCA -3’) and reverse primer (5’-TCTGCTCCATTTAGTACCGATTCTAG-3’) hybridized at positions 2048-2069 and 2091-2118, respectively, and a probe (5’- (6FAM)-AAGATCATTCTACGT-(MGB)-3’). For positive control, the same target sequence (positions 2048-2118) was cloned into into vector pUHD10.1 [69] with a forward primer (5’- CCGCGGCGGCTAAGAGT-TGCACATCCA- 3’) and reverse primer (5’-CCCGGGTCTGCTCCATTTAGTACCGATTCTAG-3’) the XmaI and SacII sites of pUHD10.1. The standard curve for each qPCR reaction plate (96 wells) was generated by 10-fold serial dilution of the pUHD10.1 positive control plasmid. The mean of triplicate measurements is shown.
