# Supplementary material for: The secreted protein Cowpox Virus 14 contributes to viral virulence and immune evasion by engaging Fc-gamma-receptors
Source: PLoS Pathog. 2022 Sep 19;18(9):e1010783. doi: 10.1371/journal.ppat.1010783 (PMC9521928; doi:10.1371/journal.ppat.1010783)
Supplement: S4 Fig — A) CPXV14 does not inhibit T cell activation by PMA/Ionomycin. Splenocytes from five female 6–10 week old BALB/cByJ mice were coincubated with virus-free supernatants from MC57 fibroblasts infected with the indicated viruses (1:1 ratio of MC57 and splenocyte SN) overnight (16 hours) at 37°C. The cells were treated with 50 ng/ml PMA, 1 μg/ml ionomycin and 4 μg/ml Brefeldin A for 6 hours at 37°C. Following stimulation the cells were washed, then stained for surface markers, followed by fixation, permeabilization and ICS for TNFα and IFNγ as shown in S1 Fig. Error bars indicate SEM. B) CPXV14 does not inhibit stimulation OT-1 T cells by SIINFEKL peptide. Splenocytes from OT-I mice (C57BL/6J background) were pre-treated with 5 μg/ml CPXV14-His (226 nM) for 4 hours and then stimulated with SIINFEKL peptide at the indicated concentrations in the presence of BFA for 6 hours. Splenocytes were surface stained and then subjected to ICS to quantitate production of OT-I specific TNFα. Plots in B show data from one representative mouse. C) Average frequency of TNFα-positive T cells of 3 mice (+SEM). The gating strategy was Lymphoctes (FSC/SSC)-> single cells -> live cells -> CD3+ -> CD8+TNFα+. (DOCX) [file ppat.1010783.s004.docx]

**
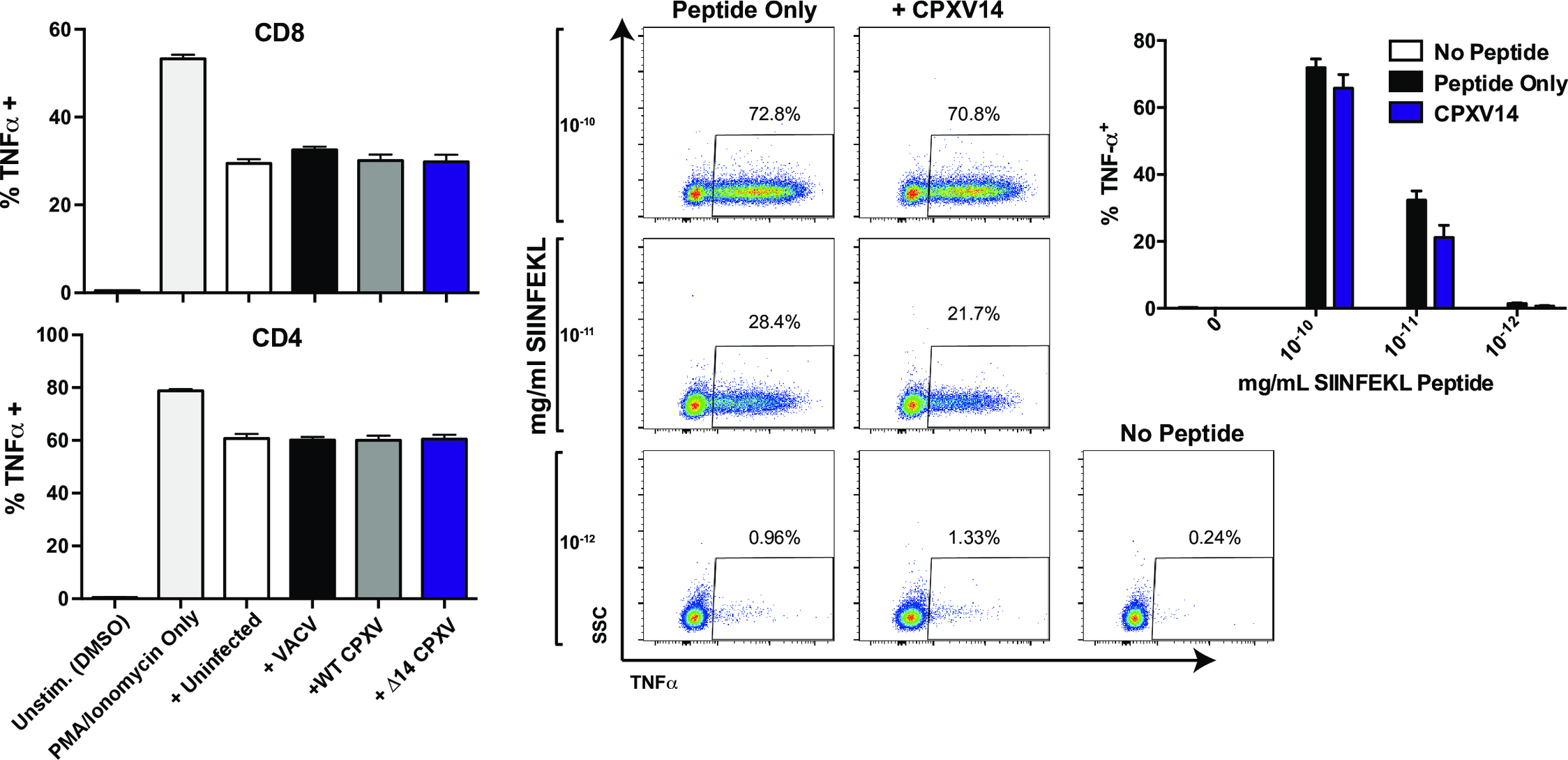
 S4 Fig. CPXV does not inhibit T cell activation by PMA/Ionomycin or peptide**

A) CPXV14 does not inhibit T cell activation by PMA/Ionomycin. Splenocytes from five female 6-10 week old BALB/cByJ mice were coincubated with virus-free supernatants from MC57 fibroblasts infected with the indicated viruses (1:1 ratio of MC57 and splenocyte SN) overnight (16 hours) at 37°C. The cells were treated with 50 ng/ml PMA, 1 μg/ml ionomycin and 4 μg/ml Brefeldin A for 6 hours at 37°C. Following stimulation the cells were washed, then stained for surface markers, followed by fixation, permeabilization and ICS for TNFα and IFNγ as shown in Fig. S1. Error bars indicate SEM.

B) CPXV14 does not inhibit stimulation OT-1 T cells by SIINFEKL peptide. Splenocytes from OT-I mice (C57BL/6J background) were pre-treated with 5 μg/ml CPXV14-His (226 nM) for 4 hours and then stimulated with SIINFEKL peptide at the indicated concentrations in the presence of BFA for 6 hours. Splenocytes were surface stained and then subjected to ICS to quantitate production of OT-I specific TNFα. Plots in B show data from one representative mouse. C) Average frequency of TNFα-positive T cells of 3 mice (+SEM). The gating strategy was Lymphoctes (FSC/SSC)-> single cells -> live cells -> CD3+ -> CD8+TNFα+.
